# Optimization of the optical transparency of bones by PACT-based passive tissue clearing

**DOI:** 10.1038/s12276-023-01089-8

**Published:** 2023-10-02

**Authors:** Byung-Ho Jin, Jiwon Woo, Mirae Lee, Seockmo Ku, Hyung Seok Moon, Seung Jun Ryu, Young-Min Hyun, Jeong-Yoon Park, Sung Uk Kuh, Yong Eun Cho

**Affiliations:** 1 The Spine and Spinal Cord Institute, Department of Neurosurgery, Gangnam Severance Hospital, Yonsei University College of Medicine, Seoul, 06273 Republic of Korea; 2 College of Medicine, Yonsei University Graduate School, Seoul, 03722 Republic of Korea; 3 Department of Neurosurgery, International ST Mary´s Hospital, College of Medicine, Catholic Kwandong University, Incheon, 22711 Republic of Korea; 4 Department of Neurosurgery, Graduate School of Medical Science, Brain Korea 21 Project, Yonsei University College of Medicine, Seoul, 03722 Republic of Korea; 5 Biomedical Research Institute, Biohedron, Seoul, 06230 Republic of Korea; 6 Biomedical Research Center, Gangnam Severance Hospital, Seoul, 06230 Republic of Korea; 7 Department of Food Science and Technology, Texas A&M University, College Station, TX 77843 USA; 8 Department of Neurosurgery, Daejeon Eulji Medical Center, Eulji University, Daejeon, 35233 Republic of Korea; 9 Department of Anatomy, Brain Korea 21 PLUS Project for Medical Science, Yonsei University College of Medicine, Seoul, 03722 Republic of Korea; 10 Department of Medical Device Engineering and Management, Yonsei University College of Medicine, Seoul, 03722 Republic of Korea; 11 Department of Neurosurgery, Wiltse Memorial Hospital, Suwon-si, Gyeonggi-do 16480 Republic of Korea

**Keywords:** Fluorescence imaging, Tissue engineering, Bone, Translational research, Bone development

## Abstract

Recent developments in tissue clearing methods such as the passive clearing technique (PACT) have allowed three-dimensional analysis of biological structures in whole, intact tissues, thereby providing a greater understanding of spatial relationships and biological circuits. Nonetheless, the issues that remain in maintaining structural integrity and preventing tissue expansion/shrinkage with rapid clearing still inhibit the wide application of these techniques in hard bone tissues, such as femurs and tibias. Here, we present an optimized PACT-based bone-clearing method, Bone-mPACT+, that protects biological structures. Bone-mPACT+ and four different decalcifying procedures were tested for their ability to improve bone tissue clearing efficiency without sacrificing optical transparency; they rendered nearly all types of bone tissues transparent. Both mouse and rat bones were nearly transparent after the clearing process. We also present a further modification, the Bone-mPACT+ Advance protocol, which is specifically optimized for processing the largest and hardest rat bones for easy clearing and imaging using established tissue clearing methods.

## Bone diseases: novel imaging protocol for large bones

An optimized imaging technique specifically designed for large, hard bones could significantly enhance evaluations of animal models of different bone diseases. To fully visualize and understand the structure of complex biological tissues, scientists use techniques known as ‘optical clearing’ to enhance the resolution and optical clarity of images. One such technique is passive clearing (PACT), which has already produced high-quality, three-dimensional analysis of multiple biological tissues. However, using PACT for large, hard bone samples is challenging and time-consuming. Now, Yong Eun Cho at Yonsei University in Seoul, South Korea, and co-workers have developed an optimized method for bone clearing called Bone-mPACT+. Their technique, which the team trialed on rat femurs and tibias, dramatically improved clearing efficiency for dense bones without losing optical transparency and image resolution.

## Introduction

Significant recent advancements in the field of tissue clearing have increased the understanding of molecular patterns and cellular circuits in various biological tissues in three-dimensional space. As opposed to traditional immunohistochemistry in frozen or paraffin sections, clear lipid-exchanged acrylamide-hybridized rigid imaging/immunostaining/in situ-hybridization–compatible tissue-hydrogel (CLARITY)-based methods enable microscopy studies of tissue architecture in intact whole tissues and organs^1,2^. We recently reported the development of two novel passive tissue clearing techniques (PACTs), process-separate PACT (psPACT) and modified PACT (mPACT, which significantly reduces required tissue processing times while improving the degree of optical transparency that is achieved)^3–5^.

A full understanding of the biological processes that shape a degenerating bone requires the study of these processes across time and, when possible, across three-dimensional space. However, processing hard bones using CLARITY-based methods has proven challenging because hard bone tissues require long clearing times, even with the harsh treatments used in the majority of tissue clearing protocols. Some studies have sought to address this issue; at present, the published methods designed specifically to clear mouse bones are uDISCO^6^, FDISCO^7^, BoneClear^8^, and Fast 3D Clear^9^ methods, all of which are known to rapidly achieve clearing.

Here, we present an optimized version of a clearing method specifically geared toward clearing rodent bones. The protocol, which we refer to as Bone-mPACT+, utilizes four decalcifying agents (20% ethylenediaminetetraacetic acid [EDTA], HCl-based Calci-Clear Rapid, 5% nitric acid, and 10% formic acid) and maintains tissue integrity without compromising the achieved optical transparency. We also present Bone-mPACT+ Advance, which allows the clearing of thin sections of hard bones from rats. The Bone-mPACT+ Advance protocol addresses the limitations imposed by the narrow 2 mm working distance of traditional confocal microscopes, which currently prevents the study of samples larger than rat bones.

The present study is a proof-of-concept investigation of bone metabolism during pregnancy and in a rat model of male osteoporosis using a combination of Bone-mPACT+ and imaging of endogenous fluorescence by Calci-Clear Rapid decalcification. The study findings suggest that optimized bone clearing is useful and can be easily applied for physiological and anatomical evaluations of a diverse assortment of experimental animal models of bone disease.

## Materials and methods

### Experimental animal ethics

This study was carried out in strict accordance with the recommendations in the Guide for the Care and Use of Laboratory Animals of the Ministry of Agriculture, Food, and Rural Affairs (MAFRA) and was approved by the Institutional Animal Care and Use Committee (IACUC) of the Yonsei University College of Medicine (#2017-0230, Date of approval: 10 March 2020, and #2015-0147; Date of approval: 1 July 2016).

### Isolation of embryos, brain, and bones

When the mouse thorax was opened, an incision was made in the right atrium of the heart. Mice were then perfused with equal volumes of cold 0.1 M phosphate-buffered saline (PBS) and 4% (PFA). Embryos and bones were then isolated using methods described previously^5,10^. Each sample was transferred to a 50 mL tube containing sufficient 4% PFA solution to cover the tissue and stored at 4 °C for 24 h.

### psPACT and mPACT of mouse brain and embryos

Each fixed sample in 4% PFA was washed for 1 h with 0.1 M PBS in a 50 mL tube and then transferred to a new 50 mL tube containing sufficient 4% acrylamide (AA; Sigma‒Aldrich, Inc., MO, USA) in 0.1 M PBS to cover the sample. After incubation at 37 °C for 24 h, the sample was covered with photoinitiator (0.25% 2,2′-azobis[2-(2-imidazolin-2-yl)propane]dihydrochloride; VA-044; Wako Chemicals USA, Inc., VA, USA) in 0.1 M PBS in a 50 mL tube and incubated at 37 °C for 6 h. The samples for psPACT and mPACT were embedded under vacuum and nitrogen gas for 10 min. The tissue was transferred to a 50 mL tube containing a sufficient volume of one of the following clearing solutions: 8S (8% sodium dodecyl sulfate [Affymetrix, OH, USA] in 0.1 M PBS, pH 8.0), 10 C (10% sodium deoxycholate [Wako Chemicals USA, Inc., VA, USA] in 0.1 M PBS, pH 8.0), 8S10C (8% SDS and 10% SDC in 0.1 M PBS, pH 8.0) and 4S5C (4% SDS and 5% SDC in 0.1 M PBS, pH 8.0) with 0.5% α-thioglycerol (+; Sigma‒Aldrich, Inc., MO, USA). The samples were then incubated with shaking at 150 rpm at 37–45 °C until the tissue cleared.

### Bone-mPACT+

Each bone sample was submerged in 4% PFA and stored at 4 °C for 24 h. Samples for Bone-mPACT+ testing were washed with PBST (0.1 M PBS plus 0.1% Triton X-100 [Sigma‒Aldrich, Inc., MO, USA]) and then submerged in decalcification solutions [Calci-Clear Rapid (National Diagnostics, GA, USA), 20% ethylene-diamine-tetraacetic acid (EDTA; Sigma‒Aldrich, Inc., MO, USA), 5% nitric acid (Duksan Pure Chemicals, Gyeonggi-do, Republic of Korea), and 10% formic acid (Samchun Chemicals, Seoul, Republic of Korea)] at 37 °C or 45 °C. For more detailed methods, see Table 1. After a 1 h wash in PBST, samples were submerged in 4% acrylamide in 0.1 M PBS at 45 °C for 6–8 h, followed by incubation in 0.25% VA-044 in 0.1 M PBS at 45 °C for 6–8 h. Samples were embedded under vacuum and nitrogen gas for 10 min each. Samples processed with the Bone-mPACT+ protocol were removed from the embedded hydrogel, transferred to 8S10C+ clearing solution with 0.5% α-thioglycerol, and shaken in a shaking incubator at 45 °C and 150 rpm until optical transparency was achieved. Samples were then treated with 25% triethanolamine (TEA; Daejung Chemicals & Metals Co., Ltd., Gyeonggi-do, Republic of Korea) at 45 °C for 2 days. The *n*RIMS solution [refractive index (RI) 1.46] was prepared by mixing 0.8 g/mL Nycodenz (Serumwerk Bernburg, Bernburg, Germany) in 30 mL of base buffer containing 0.01% (*w/v*) sodium azide (Sigma‒Aldrich, Inc., MO, USA) and 0.1% (*v/v*) Tween-20 (Sigma‒Aldrich, Inc., MO, USA) in 0.1 M PBS, pH 7.5. Samples were washed with PBST and then incubated in *n*RIMS solution at 4 °C for 1–2 days. For more details, see Fig. 2.

**Table 1:** Comparison of decalcification efficiency and clearing time of the rodent bone tissues in this study.

Bone clearing protocol			Bone CLARITY		Bone-mPACT +							
Decalcification			10% EDTA		20% EDTA		Calci-Clear Rapid		5% Nitric acid		10% Formic acid	
Clearing condition			Decalcification time	Clearing time	Decalcification time	Clearing time	Decalcification time	Clearing time	Decalcification time	Clearing time	Decalcification time	Clearing time
			Tm: 4 °C	8S	Tm: 45 °C	8S10C+	Tm: 45 °C	8S10C+	Tm: RT	8S10C+	Tm: RT	8S10C+
Mouse	Head (+Brain)		±96 h (*n* = 2)	±1020 h (*n* = 2)	±96 h (*n* = 3)	±960 h (*n* = 3)	±10 h (*n* = 3)	±960 h (*n* = 3)	8 h (*n* = 3)	±720 h (*n* = 3)	-	-
	Lumbar Vertebrae		-	-	±96 h (*n* = 5)	±72 h (*n* = 5)	±8 h (*n* = 5)	±72 h (*n* = 5)	-	-	-	-
	Fore limb	Scapula	-	-	±96 h (*n* = 5)	±48 h (*n* = 5)	±6 h (*n* = 5)	±48 h (*n* = 5)	-	-	-	-
		Humerus	-	-	±96 h (*n* = 5)	±72 h (*n* = 5)	±6 h (*n* = 5)	±72 h (*n* = 5)	±1 h (*n* = 5)	±72 h (*n* = 5)	±8 h (*n* = 5)	±72 h (*n* = 5)
		Radius-Ulna	-	-	±96 h (*n* = 5)	±72 h (*n* = 5)	±6 h (*n* = 5)	±72 h (*n* = 5)	±1 h (*n* = 5)	±72 h (*n* = 5)	±8 h (*n* = 5)	±72 h (*n* = 5)
	Hind limb	Femur	-	-	±96 h (*n* = 5)	±96 h (*n* = 5)	±6 h (*n* = 5)	±96 h (*n* = 5)	-	-	-	-
		Tibia-Fibula	±96 h (*n* = 5)	±120 h (*n* = 5)	±96 h (*n* = 5)	±96 h (*n* = 5)	±6 h (*n* = 5)	±96 h (*n* = 5)	±1 h (*n* = 5)	-	±8 h (*n* = 5)	-
		Foot	-	-	±96 h (*n* = 5)	±96 h (*n* = 5)	±6 h (*n* = 5)	±96 h (*n* = 5)	-	-	-	-
Rat	Lumbar Vertebrae		-	-	±168 h (*n* = 5)	±240 h (*n* = 5)	±2 h (*n* = 5)	±240 h (*n* = 5)	-	-	-	-
	Fore limb	Scapula	-	-	±168 h (*n* = 5)	±168 h (*n* = 5)	±8 h (*n* = 5)	±168 h (*n* = 5)	±5 h (*n* = 5)	±240 h (*n* = 5)	±40 h (*n* = 5)	±240 h (*n* = 5)
		Humerus	-	-	±168 h (*n* = 5)	±360 h (*n* = 5)	±10 h (*n* = 5)	±360 h (*n* = 5)	±6 h (*n* = 5)	±360 h (*n* = 5)	±42 h (*n* = 5)	±288 h (*n* = 5)
		Radius-Ulna	-	-	±168 h (*n* = 5)	±240 h (*n* = 5)	±10 h *(n* = 5)	±240 h (*n* = 5)	±6 h (*n* = 5)	±360 h (*n* = 5)	±42 h (*n* = 5)	±240 h (*n* = 5)
	Hind limb	Hip Bone	-	-	±168 h (*n* = 5)	±240 h (*n* = 5)	±10 h (*n* = 5)	±240 h (*n* = 5)	±8 h (*n* = 5)	±480 h (*n* = 5)	±45 h (*n* = 5)	±240 h (*n* = 5)
		Femur	-	-	±168 h (*n* = 5)	±240 h (*n* = 5)	±12 h (*n* = 5)	±480 h (*n* = 5)	±8 h (*n* = 5)	±600 h (*n* = 5)	±45 h (*n* = 5)	±360 h (*n* = 5)
		Tibia-Fibula	-	-	±68 h (*n* = 5)	± 240 h (*n* = 5)	±12 h (*n* = 5)	±480 h (*n* = 5)	±8 h (*n* = 5)	±600 h (*n* = 5)	±45 h (*n* = 5)	±360 h (*n* = 5)

8S: 8% SDS in 0.1 M PBS; 8S10C + : 8% SDS, 10% SDC and 0.5% α-thioglycerol in 0.1 M PBS.

### Bone-mPACT+ advance

After the Bone-mPACT+ embedding process, each sample was sliced sagittally with a blade or vibratome (VT-1000-S; Leica Biosystems, Wetzlar, Germany), placed in a 50 mL tube containing 20 mL of Advance (AD)-1, and gently shaken and incubated at room temperature for 30 min. The sample was then transferred to a 50 mL tube containing sufficient AD2 in 0.1 M PBS and gently shaken and incubated at room temperature for 10 min. The sample was then transferred to a coverslip and overlaid with another coverslip. After drying for 30 min, the sample was transferred to a 50 mL tube containing sufficient 8S10C+ clearing solution containing 1% α-thioglycerol and incubated with shaking at 150 rpm for 45 °C until the tissue was cleared. All samples were washed with 0.1 M PBST and submerged in 25% triethylamine (TEA) at 45 °C for 2 days. After washing PBST, the cleared tissues were incubated in *n*RIMS solution at 4 °C for 1–2 days. (For more detailed instructions, see also Fig. 2 and Supplementary Fig. 7a.).

### Other bone clearing protocols

The following tissue-clearing protocols were also used for bone tissue: Bone-CLARITY^11^, CUBIC-L/R^12^, MACS^13^, Ce3D^14^, EZ Clear^15^, Fast 3D Clear^9^, PEGASOS^16^, BABB^17,18^, Methanol BABB^19,20^, 3DISCO^21,22^, uDISCO^6^, FDISCO^7^, and BoneClear^8^ (see Supplementary Methods).

### Immunostaining and preparation for imaging

Cleared samples were incubated in PBST for 2 h and then blocked with 2% bovine serum albumin (BSA; Sigma‐Aldrich Inc., St. Louis, MO, USA) in PBST for 6 h. The samples were incubated for 24–72 h with the following primary antibodies: anti-OPG (Biorbyt, Cambridge, United Kingdom), anti-RANKL (Biorbyt, Cambridge, United Kingdom), anti-RUNX2 (Santa Cruz Biotechnology Inc., TX, USA), anti-COL-1 (Abcam Inc., Cambridge, United Kingdom), anti-COL-4 (Abcam Inc., Cambridge, United Kingdom), anti-laminin (Santa Cruz Biotechnology Inc., TX, USA) and anti-CD31 (Santa Cruz Biotechnology Inc., TX, USA). The samples were then washed three times in PBST for 24–48 h, followed by incubation with the secondary antibodies and lectin dye (Lycopersicon esculentum (Tomato) Lectin (LEL, TL), DyLight® 594; Vector Laboratories, CA, USA) in PBST for 24–72 h. Secondary antibodies were purchased as conjugates with Alexa Fluor 488/647 (Life Technologies, Darmstadt, Germany) and transferred to *n*RIMS solution in 50 mL tubes. Immunolabeled samples were washed three times with PBST for 24–72 h and stored in 5 mL *n*RIMS for 6–24 h. Each sample was incubated in *n*RIMS in a confocal dish and covered with a 24 mm diameter coverslip. Whole bone samples in *n*RIMS were sandwiched between two 24 × 60 mm coverslips and small 1-mm-thick magnets.

### Other assays

Details on the other assays used are provided in the Supplementary Methods.

## Results

### Optimization of the tissue clearing solution for tissue preservation and rapid clearing

The psPACT protocol provides rapid tissue clearing and separation of tissue embedded in a hydrogel containing 4% acrylamide (AA) and a photoinitiator (0.25% VA-044). The tissue is first incubated in 8% sodium dodecyl sulfate (SDS) clearing solutions and in a clearing solution of mPACT (modified PACT) consisting of 8% SDS with 0.25–0.5% α-thioglycerol (TG)^3–5^. The psPACT and mPACT protocols conserve the unique sample form by separating the polymerization process and they do not require removal of the embedded hydrogel from the sample surface.

PACT-based tissue clearing methods that use SDS can give rise to swelling during the clearing process of soft tissues, such as the brain, spinal cord, embryo, and several organs. We confirmed tissue damage, in the form of shrinkage, in the intact brain samples cleared using CLARITY-based protocols^23^. This is an important issue with CLARITY-based tissue clearing protocols for soft tissue and hard bone tissues, and the presence of tissue damage confirmed the need for supplementation with decreasing clearing time.

**Fig. 1:**
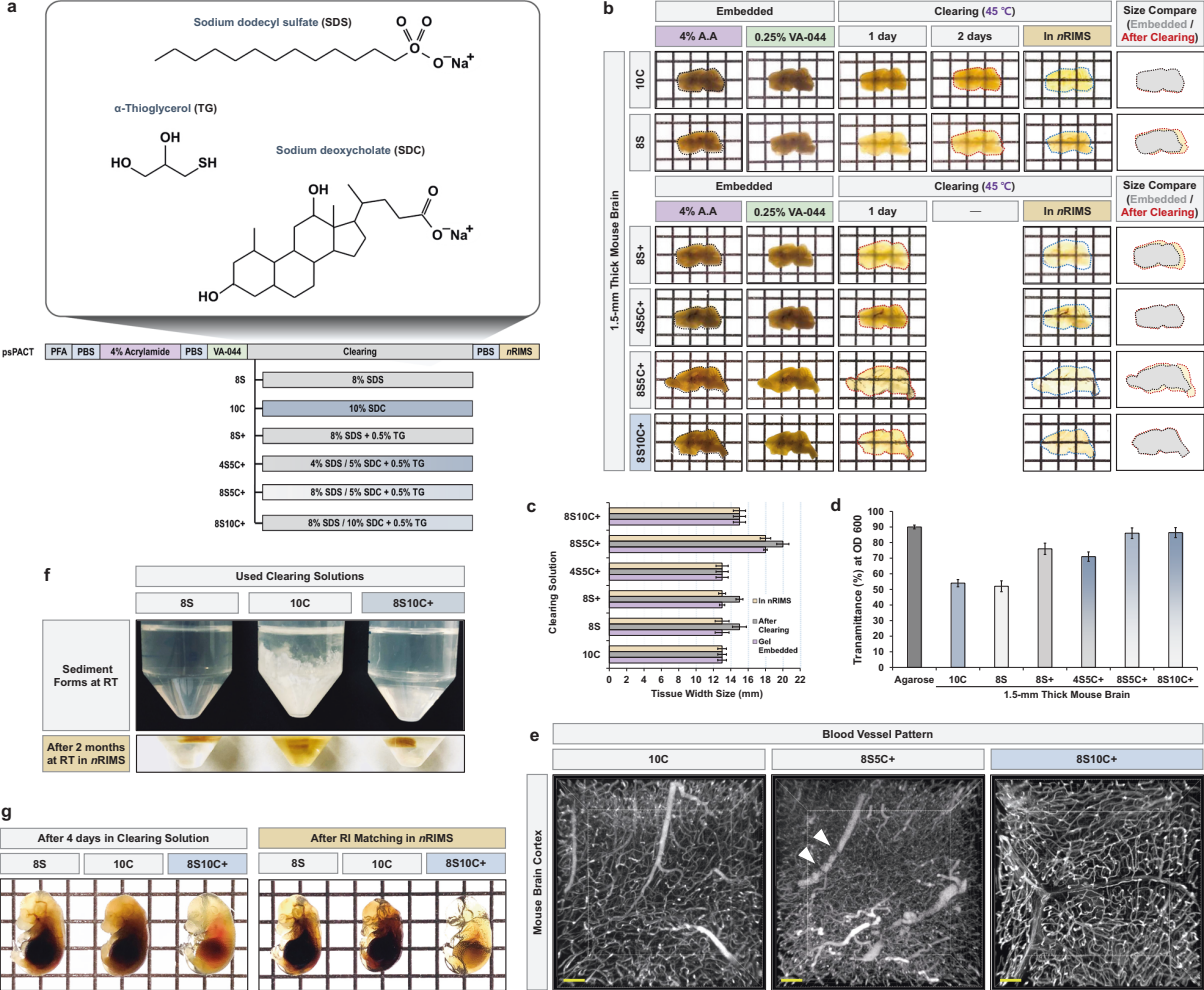
Generation of transparent mouse brain and embryos using modified PACT. **a** Schematic representation of psPACT clearing methods. Clearing solutions were produced by combining three reagents: sodium dodecyl sulfate (SDS), sodium deoxycholate (SDC), and α-thioglycerol (TG). The individual reagents or processes used for polymerization in the passive clearing methods are shown, including the various clearing solutions: 8S (8% SDS), 10S (10% SDS), 8S+ (8% SDS + 0.5% TG), 4S5C+ (4% SDS + 5% SDC + 0.5% TG), 8S5C+ (8% SDS + 5% SDC + 0.5% TG), and 8S10C+ (8% SDS + 10% SDC + 0.5% TG). **b** Comparison of optical transparency in 1.5-mm-thick mouse brain slices using the psPACT protocol and six different clearing solutions. Right images indicate the sample sizes before (embedded in 0.25% VA-044) and after clearing. Black dotted line (0.25% VA-044), red dotted line (after clearing), and blue dotted line [after refractive index (RI) matching with *n*RIMS]. **c** Comparison of brain tissue width in 1% agarose gel (1.5 mm thick) and **(b)** during the six clearing processes (each *n* = 3). **d** Comparison of transmittance (%) of cleared brain samples at OD 600 nm. The results are the averages of three separate tests. **e** Comparison of lectin immunohistochemical images of mouse brain cortex processed using psPACT with 10C, 8S5C + , and 8S10C+ clearing solutions. The lectin image for each sample was created from serial z-images (25 slices; depth: 500 μm) of the blood vessel pattern obtained by confocal microscopy at 10× magnification (0.45 NA, 2.0 mm working distance), with the microscope focused on a 1 × 1 panel (horizontal × vertical). Scale bar (yellow: 100 μm). **f** Comparison of the three tissue clearing reagents (8S, 10C, and 8S10C+) and *n*RIMS solution after incubation of each sample at room temperature. **g** Comparison of the three clearing solutions in E17.5 mouse embryos after 4 days in 8S (left), 10C (middle), and 8S10C+ (right) clearing solutions. The transparency of all the cleared samples was tested against a patterned background (length × width = 5 × 5 mm).

We prevented this problem in the present study by optimizing the tissue clearing solution. Takeyuki et al. showed that a potential clearing reagent, 10% sodium deoxycholate (SDC), increased the tissue clearing efficiency while maintaining biological structural stability during the clearing process^24^. The addition of TG to the clearing solution provided some advantages, including removal of browning and fast clearing, as previously reported for the mPACT protocol^3^. As shown in Fig. 1a, we investigated the increase in the tissue clearing efficiency of SDC by incorporating it into a PACT-based bone clearing method using clearing solutions with the following combinations of SDS, SDC, and α-thioglycerol (TG): 8S + : 8% SDS + 0.5% TG, 4S5C + : 4% SDS + 5% SDC + 0.5% TG, 8S5C: 8% SDS + 5% SDC + 0.5% TG, and 8S10C + : 8% SDS + 10% SDC + 0.5% TG.

As shown in Fig. 1b, we compared the optical transparency in 1.5-mm-thick mouse brain slices using five psPACTs and each clearing solution. Control mouse brain slices cleared in 8% SDS (8S) at 45 °C for 2 days showed swelling similar to that observed previously (after 4% PFA fixation). However, the mouse brain slices cleared in 10% SDC (10C) at 45 °C for 2 days showed no change in size during the tissue clearing process. The 10C clearing solution showed an increasing optical tissue clearing efficiency, but confirming the optical transparency was difficult because the image was more opaque than the image obtained with the 8S clearing solution.

We then compared the optical clearing transparency of mouse brain slices (each *n* = 3) using the 8S+, 4S5C+, 8S5C+ and 8S10C+ clearing solutions and the psPACT protocol. The samples in the 8S5C+ and 8S10C+ treatments were rapidly cleared in 1 day, for a reduction in time expenditure of one full day, and the observed optical clearing patterns were of high-transparency grade. The brain slice cleared with 8S10C+ had a similar size to the original sample, whereas the brain slice cleared with 8S5C+ showed tissue swelling after clearing (Fig. 1c, d).

We performed immunostaining for lectin to visualize blood vessels in intact mouse brains cleared using psPACT and in those cleared using the 10C, 8S5C+, and 8S10C+ solutions. Some penetrating vessels were severed (arrowheads) in the 8S5C+ -treated mouse brain, whereas such breakages were rarely observed at high resolution in the 8S10C + -treated mouse brain (Fig. 1e). We compared the clearing solutions after tissue clearing, and we confirmed crystal formation in the 10C sample but no crystals in the 8S10C+ sample. The 8S10C+ cleared sample also did not undergo any color change during long-term storage at room temperature in a refractive index (RI) matching [*n*RIMS: Nycodenz-based Refractive Index Matching Solution, RI: 1.46] solution (Fig. 1f).

We also compared the optical tissue clearing efficiency of 8S10C+ in mouse embryos at the E13.5 and E17.5 embryonic stages. The mouse embryos cleared rapidly in 3–4 days (E13.5: 3 days, and E17.5: 4 days) in 8S10C+ with no apparent swelling or shrinkage, and the 8S10C+ samples showed higher optical transparency than the samples cleared in the 8S and 10C solutions (Fig. 1g and Supplementary Fig. 1). These results indicate that the 8S10C+ combination of 8% SDS and 10% SDC was excellent for rapid tissue clearing and protected the tissue from harsh clearing conditions.

### Optimization of the PACT-based bone tissue clearing method used to generate transparent bones

**Fig. 2:**
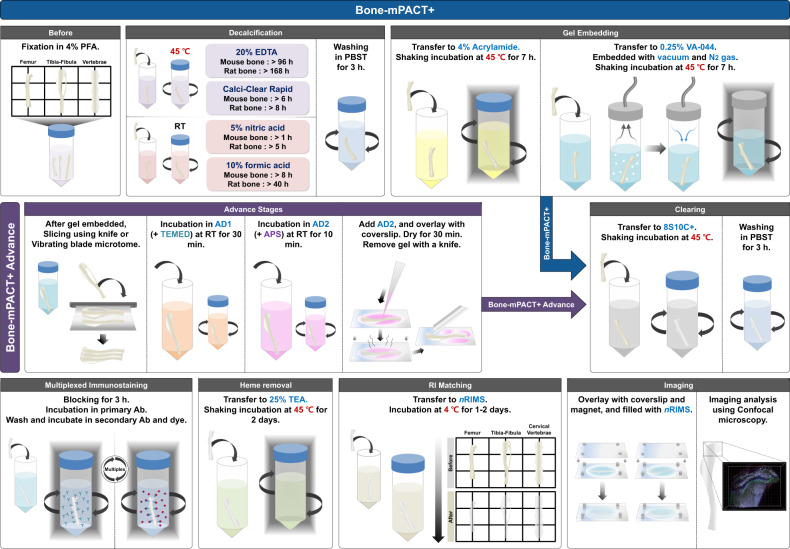
Schematic representation of Bone-mPACT+ optimized for rodent bones. The Bone-mPACT+ method involved the use of 20% EDTA, Calci-Clear Rapid, 5% nitric acid, and 10% formic acid. The method was further optimized for processing rodent bones, which require a long clearing time for tissue processing. The Bone-mPACT+ Advance method was optimized for bone marrow and protected it from swelling. Bone tissue sections larger than a mouse bone could be prepared in a 4% acrylamide-based hydrogel coating.

In this study, we optimized the PACT-based bone clearing method for three-dimensional visualization in transparent bone using a method we named “Bone-mPACT+”. This method consists of four decalcification processes. In the newly optimized Bone-mPACT+ procedure for bone tissue, the clearing and incubation steps were performed at 45 °C rather than at the standard temperature of 37 °C. This protocol yielded transparent, undamaged bone tissues with no tissue expansion or shrinkage. Figure 2 provides a detailed outline of the steps required for the Bone-mPACT+ method. In Bone-mPACT+, the bones are fixed in PFA and decalcified with decalcification solution. The bones are treated with 4% acrylamide and 0.25% VA-044, and they are embedded with vacuum and nitrogen gas (N2) followed by clearing with 8S10C+, PBS wash, prior to heme removal in 25% TEA. Bone-mPACT+ Advance follows the same steps as Bone-mPACT+, but after embedding in 0.25% VA-044, the bones are sliced into thin sections. The bones are then incubated in 4% acrylamide-based solutions (e.g., AD1 and AD2) followed by drying, clearing and PBS washing prior to heme removal in 25% triethanolamine (TEA).

**Fig. 3:**
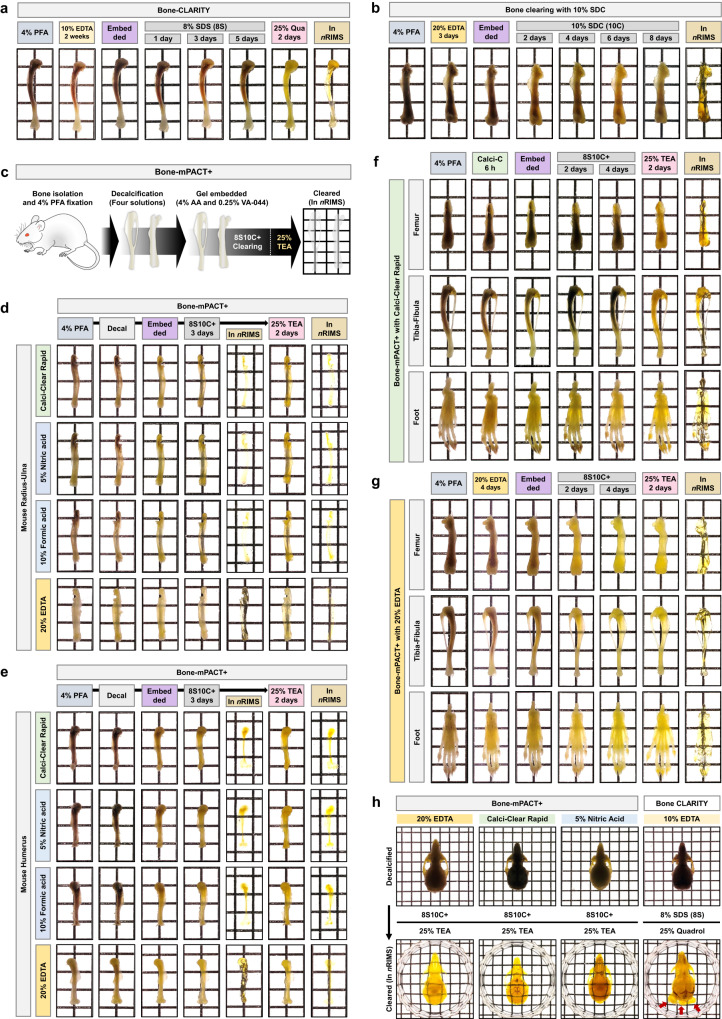
Generation of transparent mouse bones with Bone-mPACT + . **a** Optical transparency of the mouse tibia achieved with the Bone-CLARITY. **b** Optical transparency of the mouse femur using bone clearing with 10% SDC. **c** Schematic representation of the optimized Bone-mPACT+ method. **d**, **e** Comparison of optical transparency of the mouse forelimb bones (radius-ulna and humerus) achieved with the Bone-mPACT+ using Calci-Clear Rapid, 5% nitric acid, 10% formic acid, and 20% EDTA. **f**, **g** Comparison of optical transparency of the mouse hindlimb bones (femur, tibia-fibula, and foot) with Bone-mPACT+ protocols using Calci-Clear Rapid and 20% EDTA. **h** Comparison of the optical transparency of the mouse skulls (containing the brain) achieved with the Bone-PACT+ protocol using 20% EDTA, Calci-Clear Rapid, and 5% nitric acid.

The previously reported Bone-CLARITY^11^ and PACT-DeCAL^25^ protocols provide successful bone clearing, as the most important issues are the decalcification of hard bone tissue and decreased autofluorescence after clearing. Decalcification during the bone clearing protocol removes minerals from the bone to create soft bone tissue and facilitates the permeation of embedding and clearing solutions into the bone tissue. Decalcification solutions such as ethylenediaminetetraacetic (EDTA), hydrochloric acid, nitric acid, and formic acid are most commonly used in histological research^26,27^. Decalcification in 10–20% EDTA, 5% hydrochloric acid, and 5% nitric acid was slowest at low temperature (e.g., 4 °C) and fast at high temperature (e.g., 37–45 °C). However, decalcification in 10% formic acid was fast at low temperature (e.g., 4 °C). EDTA causes minimal damage to the bone tissue during the decalcification process compared with the others. The currently available bone clearing protocols use 10–20% EDTA for decalcification, but EDTA decalcification takes a very long time (Fig. 3a) (e.g., PEGASOS: 5 days at 37 °C, and Bone-CLARITY: 14 days at 4 °C)^11,16,25^. The Bone-CLARITY protocol required 2 weeks for decalcification, and a longer passive clearing time of >5 days in 8% SDS (total 3 weeks). We reduced the decalcification times for hard mouse femur bone tissue by modifying the PACT-based bone clearing protocol. As shown in Fig. 2, mouse femur bone was rapidly decalcified in 3 days with gentle shaking and incubation at 45 °C in 20% EDTA, but the bone sample required a long time (8 days) to achieve full transparency in the 10% SDC (10C) clearing solution (Fig. 3b).

A recent study showed that heme-rich bone marrow creates an autofluorescence problem, but this issue can be reduced by heme removal with a 25% solution of quadrol (*N,N,N’,N’*-tetrakis(2-hydroxypropyl)ethylenediamine), an amino alcohol, before refractive index matching^11,16,25^. Quadrol is highly viscous at room temperature; therefore, the use of this liquid has the inevitable drawbacks of requiring incubation at >50 °C and the expense of using a specific amino alcohol. We examined the possibility of using an amino alcohol, 25% triethanolamine (TEA), which is easy to use and low cost, and we compared its effectiveness at removing autofluorescence (Supplementary Fig. 2a, b). Note that TEA is incorporated at a 10% concentration in the CUBIC-R+ solution used in the CUBIC-L/R protocol^12^. We generated transparent rat thoracic vertebrae with a prototype Bone-mPACT+ and incubated the samples at 45 °C for 2 days in solutions containing TEA at 10–25%. The 25% TEA solution gave the highest optical transparency after refractive index (RI) matching in *n*RIMS solution at 4 °C for 1 day (Supplementary Fig. 2c).

Mouse femur bones incubated for 2 days in 25% quadrol and 25% TEA showed increased optical transparency to the naked eye when incubated in *n*RIMS solution for 1 day compared with samples not treated with an amino alcohol (Supplementary Fig. 2d, e). Autofluorescence imaging in the transparent mouse femur bone samples treated with 25% quadrol versus 25% TEA showed no significant differences in autofluorescence signals. We attempted to decrease the autofluorescence of bone by incubating the samples in two solutions in a bone clearing process, but this step did not have much effect on autofluorescence (Supplementary Fig. 2f). The use of Sudan Black (e.g., an autofluorescence quenching kit) has been proposed for decreasing bone autofluorescence, but its use is limited in histological analysis to thin bone slices^28^, and it does not allow for PACT-based bone clearing (Supplementary Fig. 3). Nevertheless, identifying a replacement agent for quadrol was difficult, but TEA increased the optical transparency during the bone clearing process.

### Generation of transparent rodent bones using the Bone-mPACT+ method

Our successful demonstration of the feasibility and efficiency of the 8S10C+ clearing solution and 25% TEA incubation was expanded to compare the efficacy of these optimized protocols to those of the currently established bone clearing techniques. Figure 2 shows the Bone-mPACT+ method, which included the use of four decalcification solutions (20% EDTA, Calci-Clear Rapid, 5% nitric acid, and 10% formic acid).

Four Bone-mPACT+ methods were tested after each reagent. The Bone-mPACT+ protocols were based on the psPACT protocol for the polymerization process and combined with the 8S10C+ clearing step and the 25% TEA incubation step after clearing (Fig. 3c). Finally, the bone samples were incubated in *n*RIMS for RI matching. We compared the mouse bone clearing efficiency of the Bone-mPACT+ protocols using four decalcification solutions (Fig. 3d–h). As shown in Table 1, mouse fore limb (radius-ulna and humerus) samples were rapidly decalcified in each of the decalcifying solutions at different times (20% EDTA: 4 days at 45 °C, Calci-Clear Rapid: 6 h at 45 °C, 5% nitric acid: 1 h at room temperature, 10% formic acid: 8 h at room temperature), and the samples were cleared by treatment with Bone-mPACT+ at 45 °C for 3 days with an 8S10C+ clearing solution. (Fig. 3d, e).

We also compared the mouse hind limb (femur, tibia-fibula, and foot) bones using Bone-mPACT+ with clearing using Calci-Clear Rapid or 20% EDTA. A similar comparison of the clearing of mouse hind limb bones (femur, tibia-fibula, and foot) between Bone-mPACT+ and Calci-Clear Rapid or 20% EDTA confirmed clarification in 3–4 days (Fig. 3f, g, and Supplementary Fig. 4).

**Fig. 4:**
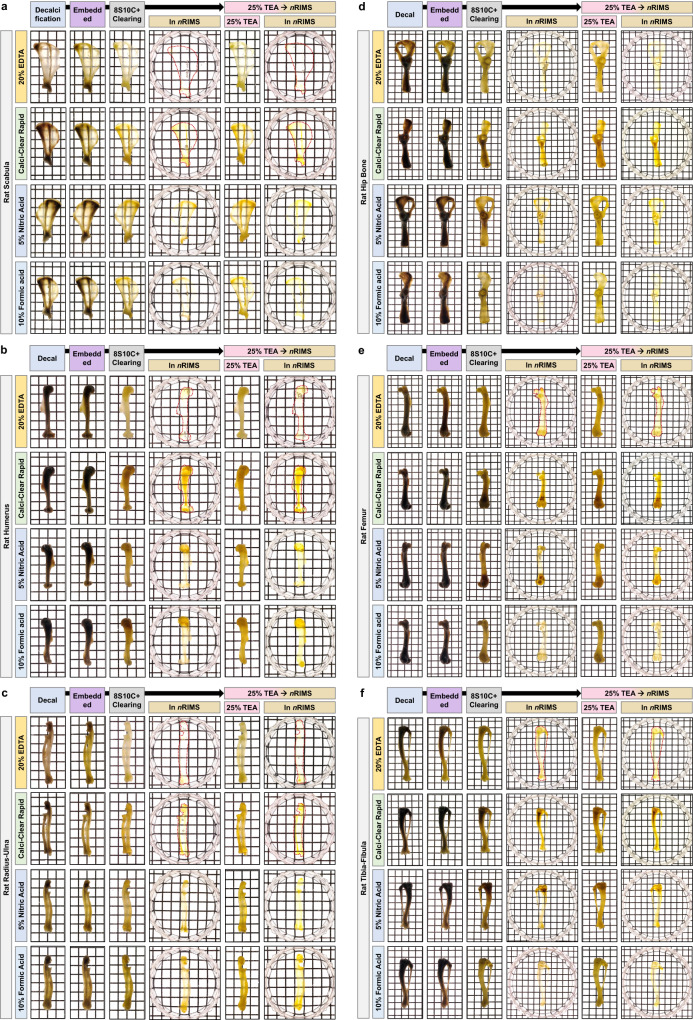
Generation of transparent rat bones with Bone-mPACT + . Optical transparency of the mouse forelimb bones (scapula, humerus, and radius-ulna) (**a**–**c**) and hindlimb bones (hip bone, femur, and tibia-fibula) (**d**–**f**) achieved with the Bone-mPACT+ using Calci-Clear Rapid, 5% nitric acid, 10% formic acid, and 20% EDTA.

We also compared the optical bone tissue clearing efficiency of Bone-CLARITY versus decalcification in 10% EDTA and with Bone-mPACT+ versus three decalcifying solutions (20% EDTA, Calci-Clear Rapid, or 5% nitric acid) for clearing mouse skulls containing brain tissue (Fig. 3h). The samples cleared with Bone-CLARITY, containing 8% SDS and 25% quadrol, were cleared within 50 days, but we observed sample damage in the form of shrinkage and swelling of the inner constructs (e.g., brain). The use of Bone-mPACT+ successfully cleared these mouse bones in 30–40 days, thereby decreasing the total bone clearing time (see Table 1). As shown in Fig. 4, the larger rat bones were successfully decalcified by Bone-mPACT+ (20% EDTA: 7 days, Calci-Clear Rapid: 8–12 h, 5% nitric acid: 5–8 h, and 10% formic acid: 40–45 h), and the samples were finally cleared in 7–25 days in 8S10C+ (see also Table 1). We also compared the transparency after RI matching (in *n*RIMS) of mouse pelvis bones after the clearing process and four decalcification treatments. We observed significant differences in transparency between decalcification and clearing, as well as across protocols, for the same region (the wing region of the ilium) of bone (Supplementary Fig. 5).

**Fig. 5:**
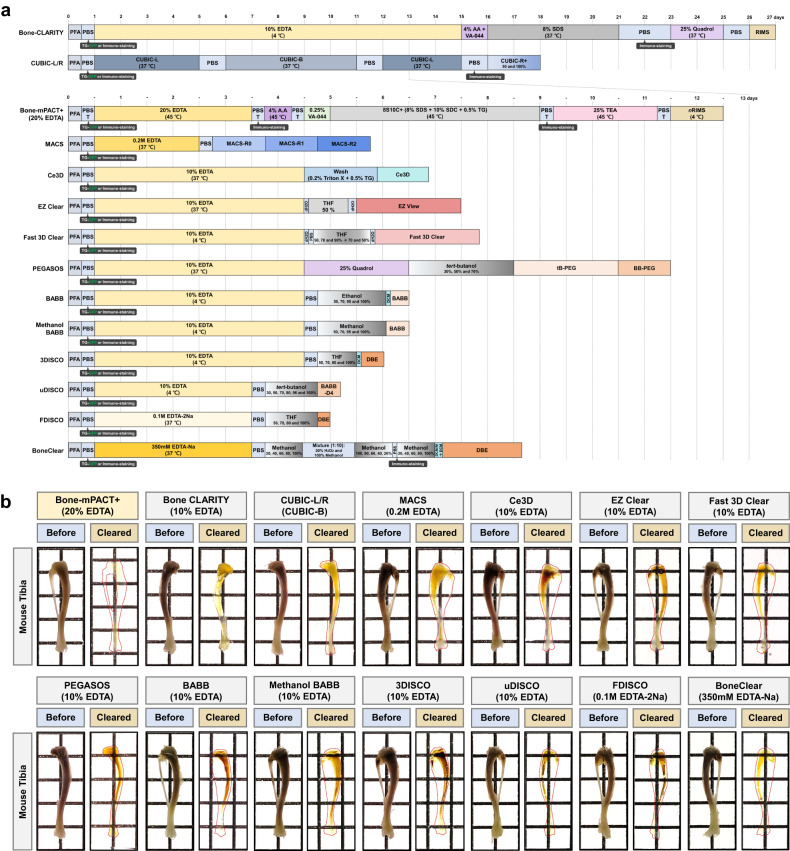
Generation of transparent mouse tibia samples by passive bone clearing methods. **a** Schematic representation and timeline of the passive bone clearing methods. The individual reagents or processes used in the fourteen clearing methods for the decalcification and clearing process are shown. **b** Comparison of optical transparency achieved in mouse tibia samples processed using Bone-mPACT+ (with 20% EDTA), Bone-CLARITY, CUBIC-L/R, MACS, Ce3D, Fast 3D Clear, EZ Clear, PEGASOS, BABB, methanol BABB, 3DISCO, uDISCO, FDISCO, and BoneClear. The transparency of all the cleared samples was evident when they were placed on a patterned background (length × width = 5 × 5 mm).

As shown in Fig. 5a, we also compared it to thirteen previously reported bone clearing protocols (e.g., Bone-CLARITY^11^, CUBIC-L/R^12^, MACS^13^, Ce3D^14^, EZ Clear^15^, Fast 3D Clear^9^, PEGASOS^16^, BABB^17,18^, Methanol BABB^19,20^, 3DISCO^21,22^, uDISCO^6^, FDISCO^7^, and BoneClear^8^) and Bone-mPACT+ with 20% EDTA. The optical transparency of the mouse tibia was higher after processing with Bone-mPACT+ than with other clearing protocols (Fig. 5b and Supplementary Fig. 6).

### Comparison of mouse bone images using Bone-mPACT+

**Fig. 6:**
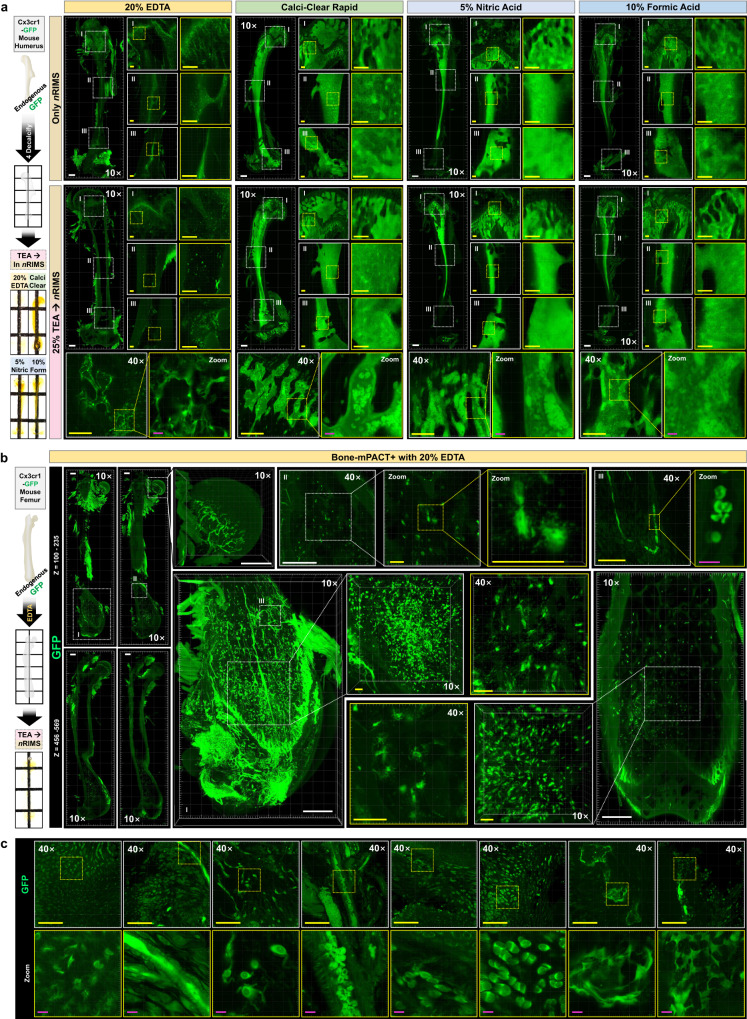
Comparison of Cx3cr1-GFP mouse bone imaging using Bone-mPACT + . **a** Comparison of GFP (green) expression patterns in Cx3cr1-GFP mouse humerus samples processed using Bone-mPACT+ with four decalcification solutions: 20% EDTA (3 × 8 tiled, range: 120–133 μm), Calci-Clear Rapid (3 × 8 tiled, range: 131–137 μm), 5% nitric acid (3 × 8 tiled, range: 134–142 μm), and 10% formic acid (3 × 8 tiled, range: 118–122 μm). Comparison of upper images (directly incubated in *n*RIMS after clearing) and lower images (incubated in 25% TEA and then *n*RIMS after clearing). **b** The GFP expression pattern in Cx3cr1-GFP mouse femur samples processed using Bone-mPACT+ with 20% EDTA. (left) The upper (*z* = 110–235 μm) and lower (*z* = 456–569 μm) images of mouse femurs were 3 × 11 tiled. A 3D projection of endogenous fluorescence (GFP) expression focusing on the distal epiphysis (2 × 3 tiled, range: 353 μm) and femoral head (2 × 2 tiled, range: 555 μm), including the bone marrow (1 × 1 tiled, range: 30 μm). **c** The morphology of GFP expressing cells in Cx3cr1-GFP mouse femurs processed using Bone-mPACT+ with 20% EDTA. All images were acquired with a 10× and 40× objective on a confocal laser microscope. Scale bar (white: 500 μm, yellow: 100 μm, magenta: 10 μm).

We investigated endogenous GFP expression in bone by generating transparent humerus bone of Cx3cr1-GFP mice^29^ using Bone-mPACT+. We also compared the endogenous GFP expression images generated by Bone-mPACT+ and four decalcifying solutions (20% EDTA, Calci-Clear Rapid, 5% nitric acid, and 10% formic acid) with incubation in *n*RIMS alone or in *n*RIMS after 25% TEA treatment (Fig. 6a). The mouse femur bones cleared with 25% TEA showed better resolution for deep imaging and increased transparency. The morphological structures of the mouse bones processed with Bone-mPACT+ plus 20% EDTA could also be observed better at higher resolutions without the bone marrow autofluorescence that occurred with the other decalcifications.

We also visualized the endogenous GFP expression of the Cx3cr1-GFP mouse femur bone after treatment with Bone-mPACT+ plus 20% EDTA (Fig. 6b). GFP-expressing cells (e.g., osteoblasts, osteoclasts, osteocytes, osteogenic cells, and blood cells) were visualized in various regions of the femur bone (Fig. 6c). The use of Bone-mPACT+ allowed visualization of molecular patterns in the mouse bones and showed the three-dimensional expression of endogenous GFP fluorescence in the bones.

**Fig. 7:**
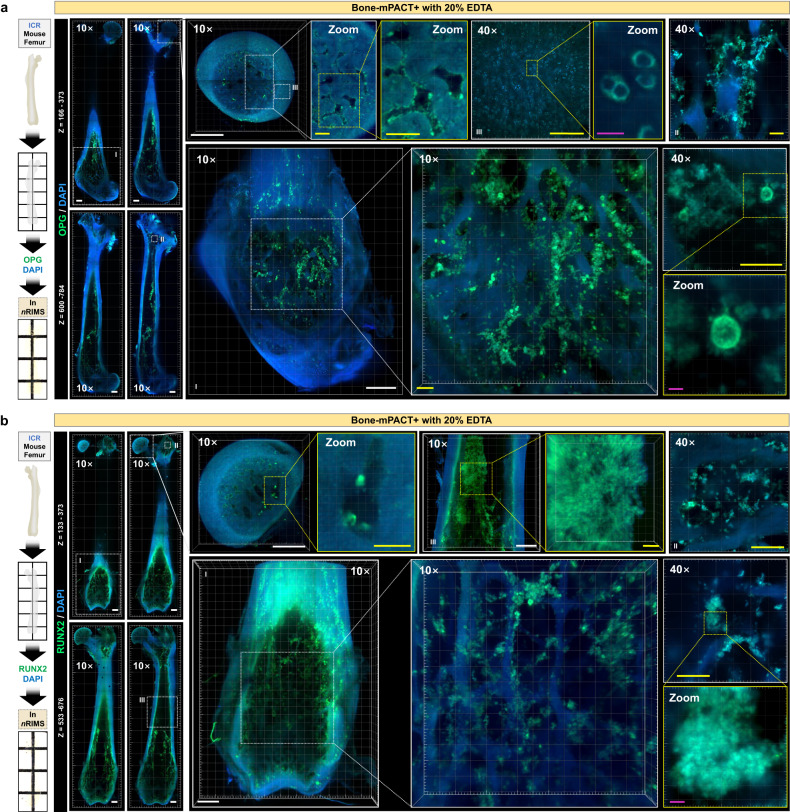
Imaging analysis of mouse bone using Bone-mPACT+. **a** OPG (green) and DAPI (blue) immunostaining in ICR mouse femur samples processed using Bone-mPACT+ plus 20% EDTA. (left) The upper (z = 166–235 μm) and lower (z = 456–784 μm) images of the mouse femur samples were 3 × 11 tiled. A 3D projection of OPG (green) expression, focusing on the distal epiphysis (3 × 3 tiled, range: 200 μm) and femoral head (2 × 2 tiled, range: 360 μm), including the bone marrow (1 × 1 tiled, range: 180 μm). **b** RUNX2 (green) and DAPI (blue) immunostaining in ICR mouse femur samples processed using Bone-mPACT+ plus 20% EDTA. Whole images (3 × 11 tiled, range: 133–676 μm) of RUNX2 (green) and DAPI (blue) expression in mouse femur bone. A 3D projection of RUNX2 (green) expression focusing on the distal epiphysis (3 × 4 tiled, range: 527 μm) and femoral head (2 × 2 tiled, range: 243 μm), including the bone marrow in the upper (2 × 2 tiled, range: 237 μm) and lower (1 × 1 tiled, range: 208 μm) images. All images were acquired with 10× and 40× objectives on a confocal laser microscope. Scale bar (white: 500 μm, yellow: 100 μm, magenta: 10 μm).

As shown in Fig. 7, immunostaining for osteoprotegerin (OPG) and runt-related transcription factor-2 (RUNX2) in mouse femur bones processed in this way revealed that OPG and RUNX2 expression was still concentrated in the bone marrow and condyle regions (e.g., the head, shaft, and distal diaphysis) that contained osteoblasts and osteoclasts (Supplementary Figs. 7 and 8). Immunostaining for COL4 (collagen type IV), COL1 (collagen type I), CD31 (blood vessel), RANKL (receptor activator of nuclear factor kappa-B ligand), and Laminin (adhesive glycoprotein) was also successful in similarly treated femur bones (Supplementary Fig. 9a). In contrast, immunostaining for RUNX2 and OPG in the mouse tibia bone processed using Bone-mPACT+ plus Calci-Clear Rapid to investigate the three-dimensional images of the bone marrow with endogenous fluorescence expression revealed strong endogenous fluorescence in the bone marrow, which complicated the immunohistochemical investigation of RUNX2 and OPG expression (Supplementary Fig. 9b).

However, using these passive bone clearing methods, we were able to visualize intact, transparent models of mouse bones, although the decalcification differed among the four methods. The optimized passive bone clearing methods achieved bone clarity. These results suggested that the Bone-mPACT+ bone clearing method that incorporates four decalcifications can generate clear bones more stably and clearly than can be achieved using current CLARITY-based passive clearing methods.

### Investigation of bone metabolism in maternal mouse bone during pregnancy using Bone-mPACT+

**Fig. 8:**
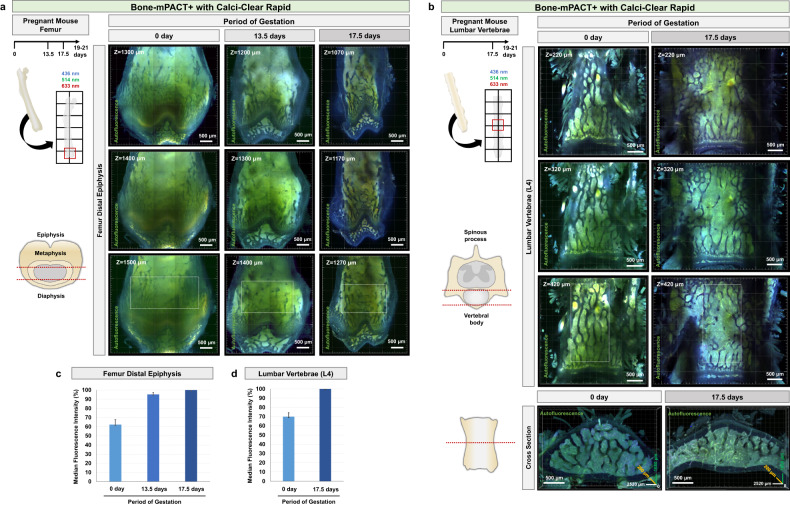
Visualization of bone marrow pattern in female mouse bones during pregnancy using Bone-mPACT+ with Calci-Clear Rapid. Comparison of endogenous fluorescence images of transparent maternal mouse femur bones (**a**) and fourth lumbar vertebrae (L4) (**b**) processed using Bone-mPACT+ with Calci-Clear Rapid. Single z-stack images of bones at (left) 0, (middle) 13.5, and (right) 17.5 gestational days (full gestation = 21 days). Scale bar (white: 500 μm). **c**, **d** Relative fluorescence intensity (bone marrow) in images of mouse femur distal epiphysis (each *n* = 5; imaging depth: 1200–1500 µm) (**a**) and mouse lumbar vertebra samples (each *n* = 5; imaging depth: 400–500 µm) (**b**). Merged images showing autofluorescence in three channels; green (wavelength: 514 nm), red (wavelength: 633 nm), and blue (wavelength: 436 nm). Each colored line indicates the values for the three distinct assessment periods. The results are the averages of five separate tests.

We applied Bone-mPACT+ to the vertebral columns of pregnant mice, as these bones are highly susceptible to fractures due to osteoporosis, and their complex geometry is particularly difficult to probe with traditional sectioning-based methods. We validated the Bone-mPACT+ protocol by applying our clearing and imaging method to investigate the marrow and trabecular patterns in biopsies obtained from the femurs and vertebrae of female mice during pregnancy. We isolated the femur and vertebrae bones from pregnant mice at 0, 13.5, and 17.5 days of gestation (mouse gestation is typically 19–21 days in duration). The femur bone tissue was transparent in a total of 10 days using Bone-mPACT+ and Calci-Clear Rapid with preserved autofluorescence (see Fig. 3f and Table 1). We visualized the endogenous fluorescence of the femur and vertebra bones, and we compared the patterns of the bone marrow (highlighted region with autofluorescence) and trabecula (dark region) of the focused condyle region in the femur (Fig. 8). We observed a decrease in the bone size and an increase in the pore size of the bone marrow, and trabecular bone loss was significantly defined at 17.5 gestational days (Fig. 8a, c). Clarification and imaging of the vertebrae from these mice, focusing on the fourth lumbar vertebral body (L4), revealed dense, opaque bone consisting mostly of cancellous bone. Similar to the femur, the lumbar vertebra also showed trabecular bone loss at 17.5 gestational days (Fig. 8b, d).

These changes in bone density suggest that pregnancy is associated with a deterioration of maternal bone mass. The metabolism of calcium resets to meet the needs imposed by the building of the fetal skeleton^30,31^. During pregnancy and lactation, the mother has an increased need for calcium to meet fetal calcium requirements^32,33^.

We also applied Bone-mPACT+ to the vertebral columns of a rat model of male osteoporosis (OCX) generated by orchiectomy^34^. The osteoporotic vertebrae are highly susceptible to fractures, and their complex geometry is again particularly difficult to probe with traditional sectioning-based methods. We cleared and imaged the vertebrae from the rat models, focusing on the second lumbar vertebral body (L2). As shown in Supplementary Fig. 10a, b, the rat vertebral column was cleared after 6 days of treatment with Bone-mPACT+ with Calci-Clear Rapid, and optical deep imaging of the vertebrae was possible after treatment with 25% TEA, which left the bones even more diaphanous. We also visualized endogenous fluorescence in the vertebrae from sham rats and at 8 weeks after orchiectomy (OCX). Comparison of the bone marrow (highlighted-region with autofluorescence) and trabeculae (dark region) confirmed a decrease in bone size and an increase in bone marrow pore size. Trabecular bone loss was especially defined at 8 weeks after orchiectomy (Supplementary Fig. 10c, d). These results provided a proof-of-concept demonstration that our Bone-mPACT+ method can facilitate three-dimensional analyses of biological structures in whole intact rodent bones.

### Development of a bone-specific bone-mPACT+ advance protocol for bone tissue clearing and retention of intact structure in large bone sections

The Bone-mPACT+ protocol successfully allowed imaging of mouse bone; however, we found that large bones and bones from larger rodents were difficult to assess due to the limitations imposed by the 2 mm working distance of the objective lens in a conventional confocal laser microscope. Large samples can be visualized using light-sheet fluorescence microscopy; however, the technique is not practical for most traditional laboratories due to limited equipment availability and high associated costs^35^. Wide applicability of the Bone-mPACT+ protocol is further limited by the long time required for processing of large bones (e.g., clearing alone requires a minimum of >10 days, followed by an additional >10 days for immunostaining).

A large, heavy bone was difficult to image using general confocal microscopy. The sample for imaging requires slicing of suitable thickness for working distance (WD; 2 mm) and then preservation of intact bone marrow in harsh clearing conditions. To address this issue, we optimized our Bone-mPACT+ method specifically for processing sections of rat bones; we named this new protocol Bone-mPACT+ Advance. Bone-mPACT+ also used an ammonium persulfate (APS) and tetramethyl ethylenediamine (TEMED)-based solution for embedding, as opposed to the VA-044 azo initiator used in the original CLARITY protocol^1^. As shown in Fig. 2, Bone-mPACT+ Advance follows the same steps as the two-step Bone-mPACT+ protocol, but after hydrogel embedding, the rat bones are sliced into 2 mm sections. The sections were then incubated in a 4% acrylamide-based solution containing TEMED (Advance (AD)-1), followed by a 4% acrylamide-based solution containing APS (AD2) prior to the second hydrogel embedding^10^. Each bone slice was transferred to a coverslip and overlaid with a second coverslip, and the bone marrow was covered with a rapidly gelling embedding hydrogel. The Bone-mPACT+ Advance method successfully achieved optical clearance of rat tibia bone sections within 5 days via 8S10C+ (decalcification times with Calci-Clear Rapid: 1 day, and clearing times with 8S10C+: 4 days), with little tissue damage (Supplementary Fig. 11a).

We provided a proof-of-concept demonstration by processing 2-mm-thick femur and tibia bone slices derived from the male rat osteoporosis model using our “Bone-mPACT+ Advance” protocols with Calci-Clear Rapid solution. We visualized the endogenous fluorescence in the femur and tibia bones of the male OCX rat model at 8 weeks after orchiectomy, and we observed the bone marrow (highlighted region with autofluorescence) and trabecular (dark region) patterns with high-resolution imaging (Supplementary Fig. 11b, c).

## Discussion

We previously described a process-separate passive clearing technique (psPACT) and a modified PACT (mPACT) that rapidly achieved optical transparency with limited equipment and minimal hands-on processing time and without a requirement for electrophoretic tissue clearing^3–5^. When applied to whole mouse CNS tissue and embryos, both psPACT and mPACT significantly improved tissue transparency compared to existing methods^5,10^. Here, we present Bone-mPACT+, an optimized PACT-based protocol for hard bone clearing that was specifically optimized to achieve optical transparency in intact rodent bones, again without the need for electrophoretic tissue clearing.

While the original CLARITY method has significantly advanced our understanding of the three-dimensional relationships between biological structures with unprecedented detail, its relatively harsh treatments are not amenable to clearing rodent bones^1^. For this reason, various methodologies have been developed specifically for mouse bone tissue, but they are not without their own limitations. For instance, Bone-CLARITY (an optimized version of PACT-deCAL) requires roughly four weeks to achieve optical transparency with its decalcification process at low temperature (i.e., 4 °C) in 10% EDTA^11,25^.

The CLARITY-based PACT tissue clearing protocol involves the use of a hybrid tissue hydrogel embedded with 4% acrylamide and photoinitiator (0.25% VA-044). The transparent tissue is finally generated with an extra incubation in 8% sodium dodecyl sulfate (SDS) clearing solution after embedding. The resulting hybrid PACT sample shows obvious tissue swelling during the clearing step with 8% SDS. The tissue damage and expansion that occurs during clearing of the hard bone tissue with these methods are major problems and create structural differences between the hard tissues and the internal bone marrow.

Hard bone tissue requires a decalcification step to remove minerals and prevent changes to soft tissue before bone clearing, and intact bone is easily decalcified by incubation in 10–20% EDTA, as previously reported^11,16,27,36,37^. Decalcification in EDTA is accelerated above 37 °C, but EDTA at room temperature or low temperature (4 °C) provides optimal results for immunohistochemistry and cellular and structural details. However, this decalcification requires a long time^27^. The Bone-mPACT+ protocol decreases the decalcification time with incubation at 45 °C in 20% EDTA, together with the use of Calci-Clear Rapid, 5% nitric acid, and 10% formic acid solutions.

Previous studies initially deemed sodium deoxycholate (SDC) not applicable for use in animal tissues^2,38^; however, SDC clearing has subsequently resulted in the best optical transparency, and it rendered the mouse brain fully transparent after RI matching with ScaleCUBIC-2^24^. In the present study, to ensure the retention of bone structural integrity and to prevent hydrogel expansion with rapid tissue clearing, we optimized the tissue clearing process of psPACT with a clearing solution (8S10C+) by mixing 8% SDS and 10% SDC with 0.5% α-thioglycerol (TG). As shown in Fig. 1b, mouse brain slices and embryos processed by psPACT with the 8S10C+ clearing solution showed rapid clearing efficiency and high optical transparency. The combination of 8% SDS and 10% SDC showed the best clearing efficiency, at least under our psPACT conditions. The average light-transmittance ratio was slightly higher for the 8S10C + -cleared tissues than for tissues cleared only with SDS or SDC.

We also removed the autofluorescence of bone cleared using Bone-mPACT+ by incorporating an additional incubation step with 25% TEA, which is a more readily available reagent and less expensive than amino alcohols such as quadrol^11,16^. As shown in Fig. 3, mouse bone tissues processed through Bone-mPACT+ with Calci-Clear Rapid (total 10 days) and 20% EDTA (total 13 days) showed greater resistance to tissue swelling after clearing. Bone-mPACT+ provides high optical transparency of both soft and hard tissues, with rapid clearing in 8S10C+ clearing solution and postincubation in 25% TEA at 45 °C. Therefore, we further optimized Bone-mPACT+ for clearing thin sections rather than large rat bones. This protocol, which we termed Bone-mPACT+ Advance, involves processing rat bone with Bone-mPACT+ up to the hydrogelation step, followed by thin slicing and subsequent incubation in AD1 and AD2. Slices of large rat bones were successfully cleared with Bone-mPACT+ Advance with minimal damage to tissue integrity.

As a proof-of-concept demonstration, we compared the endogenous GFP fluorescence expression of femur bones from a Cx3cr1-GFP mouse after clearing using Bone-mPACT+ and the four decalcification processes and then investigated GFP expression in the cells in femur bones cleared using Bone-mPACT+ with 20% EDTA. We investigated the bone marrow and trabecular pattern of femur and lumbar vertebrae bones from pregnant mice after clearing using Bone-mPACT+ with Calci-Clear Rapid. At 13.5 and 17.5 gestational days, the bone size had decreased and the bone marrow pore size had increased; trabecular bone loss was especially defined^39^. These results are similar to the observations made in the lumbar vertebrae of male OCX model rats following orchiectomy^34^. We investigated the expression of OPG (osteoprotegerin), RUNX-2 (Runt-related transcription factor-2), RANKL (receptor activator of nuclear factor kappa-B ligand), COL1 (collagen type I), COL4 (collagen type IV), and Laminin (adhesive glycoprotein), and we visualized blood vessels with CD31 staining, in mouse femur bones made transparent using Bone-mPACT+.

Importantly, these studies have demonstrated the feasibility and efficacy of using Bone-mPACT+ and its advanced version (Bone-mPACT+ Advance) for clearing bones derived from experimental animals, thereby further broadening the applicability of CLARITY-based methods for studying biological structures^11^. In addition, our results identify a powerful investigative tool for the structural characterization of bones using 3D analysis of intact bone in experimental animals. Bone-mPACT+ facilitates the rapid examination of the 3D morphological and therapeutic aspects of surgical animal disease models and can be used to aid in the investigation of medical conditions, such as serious degenerative disease, bone cancers, and malformations, as well as for surgical studies.

Bone-mPACT+ enables high-resolution imaging and immunolabeling of diverse cellular structures in mouse bones while optimizing the preservation of stable bone marrow. Despite the significant advantages of Bone-mPACT+, further testing of its histological applications is still needed. Additionally, the optical transparency achievable with CLARITY-based clearing is not as complete as that of hydrophobic tissue clearing methods (e.g., BABB, 3DISCO, iDISCO, and PEGASOS) used for rapid whole-bone clearing with organic solvents^23^. Nevertheless, Bone-mPACT+ achieves high transparency. Achieving higher transparency in CLARITY (or PACT)-based tissue clearing requires the removal of lipids through harsh treatments. Although the Bone-mPACT+ method reduces clearing time compared to Bone-CLARITY protocols, the process still needs streamlining. Future optimization of the technique should focus on reducing clearing time and simplifying the process. We anticipate that this method will readily work on similar-sized samples with mouse bone tissues. Thus, the advanced PACT-based bone clearing technique, Bone-mPACT+, is poised to serve the research field in future investigations of metabolic bone diseases.

In conclusion, our Bone-mPACT+ protocol significantly improves the optical transparency of hard bone tissues. It is faster than traditional passive clearing methods, and it requires minimal equipment and hands-on processing time. Our data suggest that our Bone-mPACT+ and Bone-mPACT+ Advance protocols could provide access to stereoscopic multiscale information that will expand the current understanding of health and disease.

### Supplementary information

Supplementary Information

## References

[1] Chung K (2013). Structural and molecular interrogation of intact biological systems. Nature.

[2] Yang B (2014). Single-cell phenotyping within transparent intact tissue through whole-body clearing. Cell.

[3] Woo J, Lee M, Seo JM, Park HS, Cho YE (2016). Optimization of the optical transparency of rodent tissues by modified PACT-based passive clearing. Exp. Mol. Med..

[4] Woo, J., Lee, E. Y., Park, H. S., Park, J. Y. & Cho, Y. E. Novel passive clearing methods for the rapid production of optical transparency In Whole CNS tissue. *J. Vis. Exp.*10.3791/57123 (2018). 10.3791/57123 PMC6101163 29806831

[5] Woo J, Kang H, Lee EY, Park S, Cho YE (2020). Investigation of PRDM7 and PRDM12 expression pattern during mouse embryonic development by using a modified passive clearing technique. Biochem. Biophys. Res. Commun..

[6] Pan C (2016). Shrinkage-mediated imaging of entire organs and organisms using uDISCO. Nat. Methods.

[7] Qi Y (2019). FDISCO: advanced solvent-based clearing method for imaging whole organs. Sci. Adv..

[8] Wang Q, Liu K, Yang L, Wang H, Yang J (2019). BoneClear: whole-tissue immunolabeling of the intact mouse bones for 3D imaging of neural anatomy and pathology. Cell Res..

[9] Kosmidis S, Negrean A, Dranovsky A, Losonczy A, Kandel ER (2021). A fast, aqueous, reversible three-day tissue clearing method for adult and embryonic mouse brain and whole body. Cell Rep. Methods.

[10] Woo, J. et al. Investigation of PRDM10 and PRDM13 expression in developing mouse embryos by an optimized PACT-Based Embryo clearing method. *Int. J. Mol. Sci.* **22**, 10.3390/ijms22062892 (2021). 10.3390/ijms22062892 PMC8000312 33809237

[11] Greenbaum, A. et al. Bone CLARITY: clearing, imaging, and computational analysis of osteoprogenitors within intact bone marrow. *Sci Transl. Med.* **9**, 10.1126/scitranslmed.aah6518 (2017). 10.1126/scitranslmed.aah6518 28446689

[12] Tainaka K (2018). Chemical landscape for tissue clearing based on hydrophilic reagents. Cell Rep..

[13] Zhu, J. et al. MACS: rapid aqueous clearing system for 3D mapping of intact organs. *Adv. Sci.* **7**, 10.1002/advs.201903185 (2020). 10.1002/advs.201903185 PMC7175264 32328422

[14] Li W, Germain RN, Gerner MY (2017). Multiplex, quantitative cellular analysis in large tissue volumes with clearing-enhanced 3D microscopy (Ce3D). Proc. Natl. Acad. Sci..

[15] Hsu, C. W. et al. EZ Clear for simple, rapid, and robust mouse whole organ clearing. *Elife* **11**, 10.7554/eLife.77419 (2022). 10.7554/eLife.77419 PMC9555867 36218247

[16] Jing D (2018). Tissue clearing of both hard and soft tissue organs with the PEGASOS method. Cell Res..

[17] McNamara, L. M. et al. Seeing through musculoskeletal tissues: improving in situ imaging of bone and the lacunar canalicular system through optical clearing. *Plos One* **11**, 10.1371/journal.pone.0150268 (2016). 10.1371/journal.pone.0150268 PMC4773178 26930293

[18] Dodt HU (2007). Ultramicroscopy: three-dimensional visualization of neuronal networks in the whole mouse brain. Nat. Methods.

[19] Stegner, D. et al. Thrombopoiesis is spatially regulated by the bone marrow vasculature. *Nat. Commun.* **8**, 10.1038/s41467-017-00201-7 (2017). 10.1038/s41467-017-00201-7 PMC5527048 28743899

[20] Gorelashvili MG, Heinze KG, Stegner D (2018). Optical clearing of murine bones to study megakaryocytes in intact bone marrow using light-sheet fluorescence microscopy. Methods Mol. Biol..

[21] Ertürk A (2012). Three-dimensional imaging of solvent-cleared organs using 3DISCO. Nat. Protoc..

[22] Courties A (2020). Clearing method for 3-dimensional immunofluorescence of osteoarthritic subchondral human bone reveals peripheral cholinergic nerves. Sci. Rep..

[23] Woo, J. et al. Comparative analyses of clearing efficacies of tissue clearing protocols by using a punching assisted clarity analysis. *Front. Bioeng. Biotechnol.* **9**, 10.3389/fbioe.2021.784626 (2022). 10.3389/fbioe.2021.784626 PMC8831720 35155401

[24] Miyawaki T (2020). Visualization and molecular characterization of whole-brain vascular networks with capillary resolution. Nat. Commun..

[25] Treweek JB (2015). Whole-body tissue stabilization and selective extractions via tissue-hydrogel hybrids for high-resolution intact circuit mapping and phenotyping. Nat. Protoc..

[26] Bogoevski K, Woloszyk A, Blackwood K, Woodruff MA, Glatt V (2019). Tissue morphology and antigenicity in mouse and rat tibia: comparing 12 different decalcification conditions. J. Histochem. Cytochem..

[27] Savi FM, Brierly GI, Baldwin J, Theodoropoulos C, Woodruff MA (2017). Comparison of different decalcification methods using rat mandibles as a model. J. Histochem. Cytochem..

[28] Schnell SA, Staines WA, Wessendorf MW (1999). Reduction of lipofuscin-like autofluorescence in fluorescently labeled tissue. J. Histochem. Cytochem..

[29] Kim, Y. R. et al. Neutrophils return to bloodstream through the brain blood vessel after crosstalk with microglia during LPS-induced neuroinflammation. *Front. Cell Dev. Biol.* **8**, 10.3389/fcell.2020.613733 (2020). 10.3389/fcell.2020.613733 PMC7753044 33364241

[30] Sanz-Salvador L, Garcia-Perez MA, Tarin JJ, Cano A (2015). Bone metabolic changes during pregnancy: a period of vulnerability to osteoporosis and fracture. Eur. J. Endocrinol..

[31] Jia H (2020). Inhibited maternal bone resorption suppress fetal rat bone development during pregnancy. Front. Cell Dev. Biol..

[32] Kovacs CS, Kronenberg HM (1997). Maternal-fetal calcium and bone metabolism during pregnancy, puerperium, and lactation. Endocr. Rev..

[33] Winter EM (2020). Pregnancy and lactation, a challenge for the skeleton. Endocr. Connect.

[34] Ryu SJ (2016). Changes in bone metabolism in young castrated male rats. Yonsei Med. J..

[35] Gomez-Gaviro MV (2017). Optimized CUBIC protocol for three-dimensional imaging of chicken embryos at single-cell resolution. Development.

[36] Liu H (2017). Evaluation of decalcification techniques for rat femurs using HE and immunohistochemical staining. Biomed. Res. Int..

[37] Ramirez, T. et al. Comparison of methods for the histological evaluation of odontocete spiral ganglion cells. *Animals (Basel)* **10**, 10.3390/ani10040683 (2020). 10.3390/ani10040683 PMC7222732 32295193

[38] Susaki EA (2014). Whole-brain imaging with single-cell resolution using chemical cocktails and computational analysis. Cell.

[39] Roberts BC (2019). The longitudinal effects of ovariectomy on the morphometric, densitometric and mechanical properties in the murine tibia: a comparison between two mouse strains. Bone.

