# Supplementary material for: Optimization of the optical transparency of bones by PACT-based passive tissue clearing
Source: Exp Mol Med. 2023 Oct 2;55(10):2190–204. doi: 10.1038/s12276-023-01089-8 (PMC10618275; doi:10.1038/s12276-023-01089-8)
Supplement: Supplementary file 1 — Supplementary Information [file 12276_2023_1089_MOESM1_ESM.pdf]

SUPPLEMENTARY INFORMATION

Title: Optimization of the optical transparency of bones by PACT-based passive tissue clearing

Authors: Byung-Ho Jin*, Jiwon Woo*, Mirae Lee, Seockmo Ku, Hyung Seok Moon, Seung Jun Ryu, Young-Min Hyun, Jeong Yoon Park, Sung Uk Kuh, and Yong Eun Cho¶

* Byung-Ho Jin & Jiwon Woo. These authors contributed equally to this work.

¶ **Corresponding:**

Yong Eun Cho, MD., Ph.D., E-mail: ydnscho@gmail.com

SUPPLEMENTARY METHODS

Experimental Animal Ethics

All animal procedures were conducted under veterinarian supervision according to the guidelines imposed by the Ethical Committee. Mice used in these studies were cared for in accordance to the National Institutes of Health "Guide for the Care and Use of Laboratory Animals" and ARRIVE guidelines.

Animal experiments

Adult male and female Institute of Cancer Research (ICR) mice (n=40) and C57BL/6 mice (n=20) were purchased from Orient Inc. (Gyeonggi-do, Korea) and were raised in a specific pathogen-free (SPF) environment. Male Cx3cr1-GFP mice (n=2) were obtained from The Jackson Laboratory (Bar Harbor, ME, USA). Mouse embryos were isolated from embryonic day 13.5 (E13.5) to E17.5. Bones from pregnant female ICR mice (n=10) were isolated from E13.5 to E17.5 (i.e., gestational days 13.5 and 17.5). Male SD (Sprague-Dawley) rats (n=20) were purchased from Central Lab Animal Inc. (Seoul, Korea), and acclimated separately in pathogen-free ventilated cages with a controlled environment (temperature 22±4℃, humidity 65±5%, day-night cycle 06:00–18:00).

The rats were permitted free intake of tap water and standard rodent chow (SAFE, Augy, France) containing 8 g/kg calcium, 4.2 g/kg phosphorus, and 1000 UI/kg vitamin D3. Male rats were orchiectomized to induce male osteoporosis, and a few control rats underwent a sham operation. All rats in the orchiectomy group underwent bilateral

orchiectomy. Those in the sham surgery (sham) group underwent the same procedure, except that the testes were merely identified, not removed. At postoperative date (POD) 8 weeks, orchiectomized rats were sacrificed using CO_2 inhalation, and their femurs and lumbar spines were removed. Each sample was transferred to sufficient 4% paraformaldehyde (PFA) solution to cover the tissue in a 50 mL tube and stored at 4 °C for 24 h.

Orchiectomy and sham operation procedure

Anesthesia was induced with 5% isoflurane, and Rompun (2.5 mg/kg, Bayer Korea, Seoul, Korea) and Zoletil (5 mg/kg, Virbac Korea, Seoul, Korea) were then injected intraperitoneally for generalized anesthesia. Anesthesia was maintained by the administration of 2.5% isoflurane and oxygen via a coaxial nose cone.

Bilateral orchiectomy was performed via a scrotal approach. The anesthetized rat was placed supine on the operating table, and its position was fixed using adhesive tape. The scrotal hair was bilaterally shaved, and betadine preparation was applied as an aseptic maneuver. If the cremaster muscle was stimulated during the betadine preparation, resulting in ascension of the testes, a downward stroke was performed to lower the testes back into place. A small 1.0-cm median incision was made through the skin at the tip of the scrotum. The cremaster muscles were opened with an incision. At the entrance to the scrotal cavity, the testicular fat pad was located with the testis, followed by the caput epididymis, the vas deferens, and the testicular blood vessels, all of which were pulled through the incision using blunt forceps. After identifying the testis, a single ligature was placed on the spermatic cord around the vas deferens and the blood vessels. The testis and epididymis were removed. This procedure was repeated for the other testis and epididymis. The cremaster muscle and scrotal skin

were sutured layer by layer. The same preparation was performed on animals in the sham operation group, allowing the authors to visually identify each testis, epididymis, vas deferens, and testicular blood vessels. After visual identification, the cremaster muscle and skin were sutured without ligation or resection of the testes or epididymis.

Other bone clearing protocols

1) Bone-CLARITY

Fixed samples in 4% PFA were washed with 0.1 M PBS and then submerged in 10% EDTA solution at 4 °C for 2 weeks with gentle shaking. Samples were submerged in cold A4P0 solution (4% acrylamide and 0.25% VA-044 in 0.1 M PBS) at 4 °C for 24 h, followed by incubation in fresh A4P0 solution at 37 °C for 6 h. Samples were embedded under vacuum and nitrogen gas for 10 min each. Samples were removed from the embedded hydrogel and transferred to 8% SDS solution in a shaking incubator at 45 °C and 150 rpm for 5 days. Sample were washed for 2 days with 0.1 M PBS and submerged in 25% (v/w) *N,N,N',N'*-tetrakis(2-hydroxypropyl)ethylenediamine (quadrol; Sigma-Aldrich Inc., St. Louis, MO, USA) in 0.1 M PBS (pH 9) at 37 °C for 2 days. Sample were washed for 1 day with 0.1M PBS and incubated in *n*RIMS solution at room temperature for 1 day.

2) CUBIC-L/R

After fixation in 4% PFA, samples were immersed in CUBIC-L solution (Tokyo Chemical Industry Co., Ltd., Tokyo, Japan) at 37 °C for 4 days in a shaking incubator. Samples were washed in 0.1 M PBS for 24 h and incubated in CUBIC-B solution (Tokyo Chemical Industry Co., Ltd., Tokyo, Japan) at 37 °C for 5 days. Samples were

washed in 0.1M PBS at 37 °C for 24 h and re-incubated in CUBIC-L solution for 3 days. Samples were washed in PBS for 24 h and pre-treated in 50% (v/v) CUBIC-R+ solution (Tokyo Chemical Industry Co., Ltd., Tokyo, Japan) in 0.1 M PBS at 37 °C for 1 day. Samples were then incubated in 100% CUBIC-R+ solution at 37 °C for 1 day.

3) MACS

Fixed samples in 4% PFA were incubated for 24 h in 0.2 M EDTA, and sample were serially incubated for 24–36 h in MACS-R0 [20% (v/v) m-Xylylenediamine (m-XDM; Sigma-Aldrich Inc., St. Louis, MO, USA) and 15% (w/v) D-sorbitol (Sigma-Aldrich Inc., St. Louis, MO, USA) in dH_2O], MACS-R1 [40% (v/v) m-XDA and 30% (w/v) D-sorbitol in 0.1M PBS], and MACS-R2 [40% (v/v) m-XDA and 50% (w/v) D-sorbitol in dH2O] solutions in 50 mL conical tubes with gentle shaking.

4) Ce3D

Fixed bone samples in 4% PFA were washed with 0.1M PBS, and decalcified in 10% EDTA for 96 h at 37 °C. The sample were incubated in wash buffer [0.2% (v/v) Triton X-100 and 0.5% (v/v) α-thioglycerol in 0.1M PBS] for 30–60 min at 37 °C. Subsequently bone sample were incubated in washing buffer for 8 h at 37 °C and again for 24–36 h at room temperature. The washing buffer was replaced every 10–14 h. Bone samples were submerged in fresh Ce3D clearing solution [22% (v/v) N-methylacetamide (Sigma-Aldrich Inc., St. Louis, MO, USA), 0.8 g/ml Nycodenz (iohexol), 0.1% (v/v) Triton X-100 and 0.5% (v/v) α-thioglycerol in 0.1M PBS]. Samples were incubated for 24 h at room temperature until they achieved optical transparency.

5) BABB

Fixed bone samples in 4% PFA were washed in 0.1M PBS and decalcified in 10% EDTA for 96 h at 37 °C. Samples were then washed in 0.1M PBS, and sequentially incubated in 25, 50, 80, and 100% (v/v) ethanol (Merck Millipore, Burlington, MA, USA) for 8 h each at room temperature. After alcohol dehydration, the samples were incubated in fresh solutions of either 100% ethanol for 12 h at room temperature. Subsequently, samples were incubated in dichloromethane (DCM; Sigma-Aldrich Inc., St. Louis, MO, USA) for 1 h at room temperature. Samples were then incubated in BABB solution [1 volume of benzyl alcohol (BA; Duksan Pure Chemicals, Gyeonggi-do, Korea) to 2 volumes of benzyl benzoate (BB; Sigma-Aldrich Inc., St. Louis, MO, USA)] for 1–2 days until they achieved optical transparency.

6) Methanol-BABB

Fixed bone samples in 4% PFA were washed in 0.1M PBS and decalcified in 10% EDTA for 96 h at 4 °C. Samples were then washed in 0.1M PBS, sequentially incubated in 50, 70, 95 and 100% (v/v) methanol (Merck Millipore, Burlington, MA, USA) for 30 min each at room temperature and stored in 100% methanol for 12 h at 4 °C. Samples were then incubated in BABB solution for 1–2 days until they achieved optical transparency.

7) 3DISCO

Fixed bone samples in 4% PFA were washed in 0.1M PBS and decalcified in 10% EDTA for 96 h at 4 °C. Samples were then washed in 0.1M PBS, and sequentially incubated in 50, 70, 80, and 100% (v/v) tetrahydrofuran (THF; Daejung Chemical & Metals Co. Ltd., Gyeonggi-do, Korea) in dH_2O for 2–12 h each at room temperature, followed by incubation in DCM for 2 h at room temperature, and then in dibenzyl ether

(DBE; Sigma-Aldrich Inc., St. Louis, MO, USA) for 1–2 days at room temperature until they achieved optical transparency.

8) uDISCO

Fixed bone samples in 4% PFA were washed in 0.1M PBS, and decalcified in 10% EDTA for 72 h at 4 °C. Samples were then washed in 0.1M PBS, sequentially incubated in 30, 50, 70, 80, 90, 96, and 100% (v/v) *tert*-butanol (Daejung Chemicals & Metals Co., Ltd., Gyeonggi-do, Republic of Korea) in dH_2O for 2–12 h at 35 °C, and incubated in DCM for 2 h at room temperature. The sample were then incubated in BABB-D4 for 12 h at room temperature, until they achieved optical transparency. BABB-D4 was prepared by mixing BABB with diphenyl ether (DPE; Daejung Chemical & Metals Co. Ltd., Gyeonggi-do, Korea) at a ratio of 4:1 and adding 0.4% (v/v) DL-alpha-tocopherol (Sigma-Aldrich Inc., St. Louis, MO, USA).

9) FDISCO

Fixed bone samples in 4% PFA were washed in 0.1M PBS, and decalcified in 0.1M EDTA-2Na for 72 h at 37 °C. Samples were then washed in 0.1M PBS, and sequentially incubated in 50, 70, 80, and 100% (v/v) THF in dH_2O for 3–12 h each at 4 °C, followed by incubation in DBE for 2 h at 4 °C until optical transparency was achieved.

10) BoneClear

Fixed bone samples in 4% PFA were washed in 0.1M PBS, and decalcified in 350 mM EDTA-Na (pH 6.5) at 37 °C for 72 h. The sample were washed in 0.1M PBS, and embedded in 0.8% agarose in 0.1M PBS. The sample were sequentially incubated in

20, 40, 60 and 80% (v/v) methanol in dH_2O for 2 h each, and then incubated in 100% methanol for 4 h at room temperature. The samples were decolorized with a mixture (v:v = 1:10) of 30% H_2O_2 (hydrogen peroxide; Samhyun Pharm, Incheon, Korea) and 100% methanol at 4 °C overnight. The samples were then incubated with an inverse gradient of 100, 80, 60, 40, and 20% (v/v) methanol in dH_2O for 2 h each, and incubated in 0.1M PBS for 2 h at room temperature. The samples were sequentially incubated in 20, 40, 60 and 80% (v/v) methanol in dH_2O for 2 h each and then incubated in 100% methanol at room temperature for 4 h. The samples were then incubated in a mixture (v:v = 2:1) of DCM and 100% methanol (2:1, v/v) twice for 2 h, followed by four incubations in 100% DCM for 30 min at room temperature. The sample were incubated in DBE for 36 h (12 h three times) at room temperature until they achieved optical transparency.

11) PEGASOS

After fixation in 4% PFA, the samples were incubated in 20% EDTA at 37 °C for 4 days. Samples were incubated in decolorization solution [25% (v/v) quadrol in 0.1 M PBS] for 48 h at room temperature. Samples were then sequentially incubated in 30, 50, and 70% (v/v) *tert*-butanol in dH_2O for 48 h, and subsequently dehydrated in tB-PEG solution [70% (v/v) *tert*-butanol, 27% (v/v) ethylene glycol (PEG; Sigma-Aldrich, Inc., MO, USA), and 3% (v/v) quadrol] at room temperature for 48 h. Processed samples were immersed in BB-PEG solution [75% (v/v) benzyl benzoate (BB) and 25% (v/v) ethylene glycol in 0.1 M PBS] for 24 h at room temperature until optical clearance was achieved.

12) Fast 3D Clear

Fixed bone samples in 4% PFA were washed in 0.1M PBS, and decalcified in 10% EDTA for 96 h at 4 °C. Samples were then washed in 0.1M PBS for 50 min, and then in dH_2O for 30 min at room temperature. The samples were incubated in 50% (v/v) THF in dH_2O with 0.01% triethylamine (Samchun Pure Chemical, Gyeonggi-do, Korea) for 1 h at 4 °C in 70% (v/v) THF in dH_2O with 0.015% triethylamine for 1 h at 4 °C, and then in 90% (v/v) THF in dH_2O with 0.03% triethylamine for 16 h at 4 °C. The sample were then incubated in 70% (v/v) THF in dH_2O with 0.015% triethylamine for 1 h at 4 °C, and then in 50% (v/v) THF in dH_2O with 0.01% triethylamine for 1 h at 4 °C. The samples were washed in dH_2O for 50 min, and then incubated in Fast 3D Clear solution (RI= 1.553) for 2 days at room temperature until they achieved optical transparency. The Fast 3D Clear solution was prepared by mixing 4.8 g/mL Nycodenz, 0.06 g/mL diatrizoic acid (Tokyo Chemical Industry Co. Ltd., Tokyo, Japan), 0.1 g/mL N-methyl-D-glucamine (Tokyo Chemical Industry Co. Ltd., Tokyo, Japan) in 40 ml of base solution containing 20% (w/v) urea (Georgiachem, Georgia, GA, USA) and 0.02% (w/v) sodium azide (optional) in dH_2O.

13) EZ Clear

Fixed bone samples in 4% PFA were washed in 0.1M PBS for 6 h, and decalcified in 10% EDTA for 96 h at 4 °C. The samples were then washed in dH_2O, and incubated in 50% (v/v) THF (Sigma-Aldrich, Inc., MO, USA) in dH_2O for 16 h at room temperature. The samples were washed in dH_2O at room temperature for 4 h. Samples were submerged in EZ view mounting solution (RI= 1.545) for 24 h at room temperature until optical clearance was achieved. The EZ view mounting solution was prepared by mixing 80% (w/v) Nycodenz, 7 M urea, and 0.05% (w/v) sodium azide in 0.02 M

sodium phosphate buffer (pH 7.4). The dissolved solution was filtered through a 0.2 μm syringe filter (Advantec; Tokyo Roshi Kaisha, Ltd., Tokyo Japan).

Autofluorescence quenching test

For autofluorescence quenching (AQ) mixture, add equal volumes of AQ-1 [1% phosphomolybdic acid (Sigma-Aldrich, Inc., MO, USA) in 10% ethanol (Merck Millipore, MA, USA)] and AQ-2 [3% sodium ascorbate (Samchun Pure Chemical, Gyeonggi-do, Republic of Korea) and 1% sodium metabisulfite (sodium disulfite; Junsei Chemical Co., Ltd., Tokyo, Japan) in H_2O] in 50 mL tube. Add AQ-3 (0.1% sodium azide in H_2O) to mixture and mixing again. AQ mixture were prepared with manual for Autofluorescence Quenching Kit (Vector TrueVIEW; Vector Laboratories, Burlingame, CA, USA). The sample was transferred to sufficient 4% PFA solution to cover the tissue in a 50 mL tube and stored at 4 °C for 24 h. The sample were washed with 0.1M PBS and then submerged in decalcification solution of Calci-Clear Rapid solution at 45 °C for 6 h. Sample was submerged in AQ mixture at room temperature for 12 h, and incubated with 30% ethanol. The sample was washed for 1 h with 0.1M PBS in a 50 mL tube and then transferred to sufficient enough 4% acrylamide in 0.1M PBS to cover the sample in a 50 mL tube at 45 °C for 24 h. Next, the sample was covered with 0.25% VA-044 in 0.1M PBS in a 50 mL tube at 45 °C for 6 h. The sample were embedded with vacuum and nitrogen gas for each 10 min. Sample was submerged in AQ mixture at room temperature for 12 h. The tissue was transferred to a 50 mL tube containing sufficient 8S+ clearing solution with 0.5% α-thioglycerol. The sample was then incubated with shaking at 150 rpm for 37 °C until the tissue cleared.

Immunohistochemistry and histological staining

Mouse bones for immunohistochemistry (IHC) were decalcified in 20% EDTA for 96 h at 45°C, washed in 0.1M PBS, paraffin-embedded, and sectioned. After deparaffinizing and rehydrating, the sections were incubated with primary antibodies: anti-OPG, anti-RANKL, anti-RUNX2, anti-COL-4, or relevant isotype control antibodies using the EnVision™ Detection System (DAKO, Glostrup, Denmark). Stained samples were analyzed using an Olympus BX50 microscope at ×20 and ×40 magnification and Olympus CellSens standard software 7.1 (Olympus).

Image processing

All clear images were captured using a digital camera (iPhone-X; Apple Inc., CA, USA) and a stereoscopic microscope (SMZ745T; Nikon, Tokyo, Japan). Confocal microscopy was performed with an LSM-780 confocal microscope (Carl Zeiss, Oberkochen, Germany) at 10× magnification (0.45 NA, 2.0 mm working distance) using the associated Zeiss software (ZEN 3.2 black edition; Carl Zeiss, Oberkochen, Germany). Three-dimensional images and videos were edited into serial images using Imaris v8.01 software (Bitplane, Belfast, United Kingdom).

Liquid viscosity test

All reagents were suctioned for 3 s at room temperature using a 10 mL serological pipette (D-51588; Sarstedt, Inc., Nümbrecht, Germany) and a Pipet-Aid® pipette pump (4-000-100; Drummond Scientific, Broomall, PA, USA). The volume of suctioned liquid was determined in mL in a 10 mL pipette. The results are the averages of five independent tests with each new pipette. The test reagents were 100% ethanol, triethanolamine (TEA), *N,N,N',N'*-tetrakis(2-hydroxypropyl)ethylenediamine (quadrol), glycerol (Junsei Honsha Co., Ltd., Tokyo, Japan), triton-X (Sigma-Aldrich Inc., St.

Louis, MO, USA), thiodiglycol (Samchun pure chemical, Gyeonggi-do, Korea) and 0.1M PBS.

Quantification of autofluorescence intensity

Quantification of autofluorescence intensity was performed using Zeiss ZEN-2 software. Fluorescence intensity as a function of imaging depth was determined by measuring the median signal intensity at z-stack imaging depths. The autofluorescence signals of single z-stack images were measured as integrated density, using ImageJ software [National Institutes of Health (NIH), MD, United States]. The mean values of the autofluorescence signal of single z-stack images were calculated. The median fluorescence intensity (%) and integrated density (%) were normalized to the intensity of the most superficial slide.

Light transmittance measurements (PACA-Light)

Sagittal sections (1.5 mm thick) of mouse brains cleared with psPACT and different combinations of clearing reagents were used to generate 6 mm round brain tablets containing the prefrontal cortex and basal ganglia in a metal eyelet, and the RI was adjusted. The bone tissues of after decalcify and clearing in Bone-mPACT+ were used to generate 3.5 mm round bone tablets in a metal eyelet. The bone tissue tablets were transferred to a 96-well dish, and a spectrometer (Molecular Devices, CA, USA) was used to measure light transmittance (%) at 600 nm (optical density). The results were analyzed with SoftMax Pro 5 software (Molecular Devices, CA, USA). An average of three measurements was calculated for each sample. The final transmittance (%) of the sample was normalized to the transmittance of the blank values (*n*RIMS solutions).

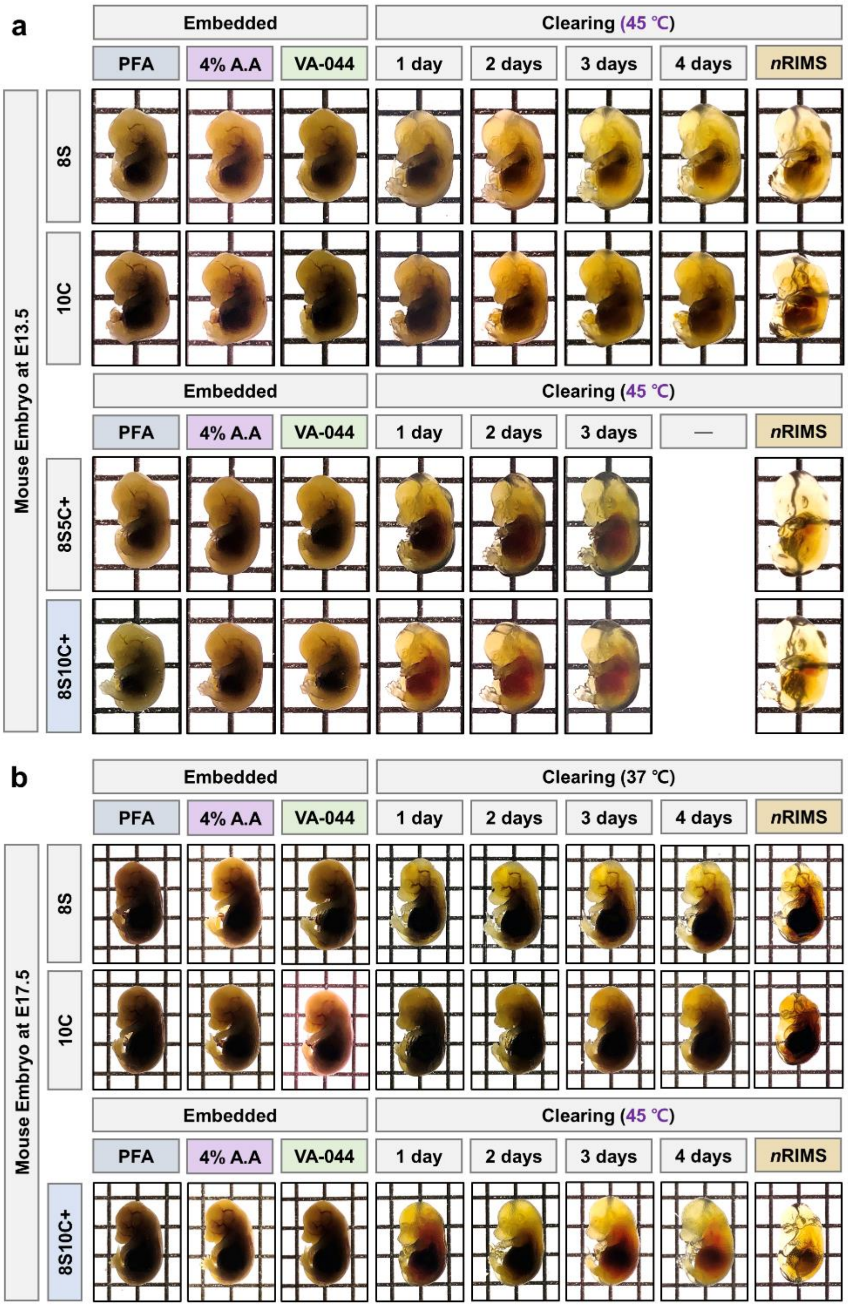

Supplementary Fig. 1. Comparison of the optical transparency of mouse embryos using various clearing solution.

Comparison of optical images in E13.5 **(a)** and E17.5 **(b)** mouse embryos using three or four clearing solutions (8S, 10C, 8S5C+ and 8S10C+) in the psPACT clearing process. The transparency of all cleared samples was tested against a patterned background (length × width=5 × 5 mm).

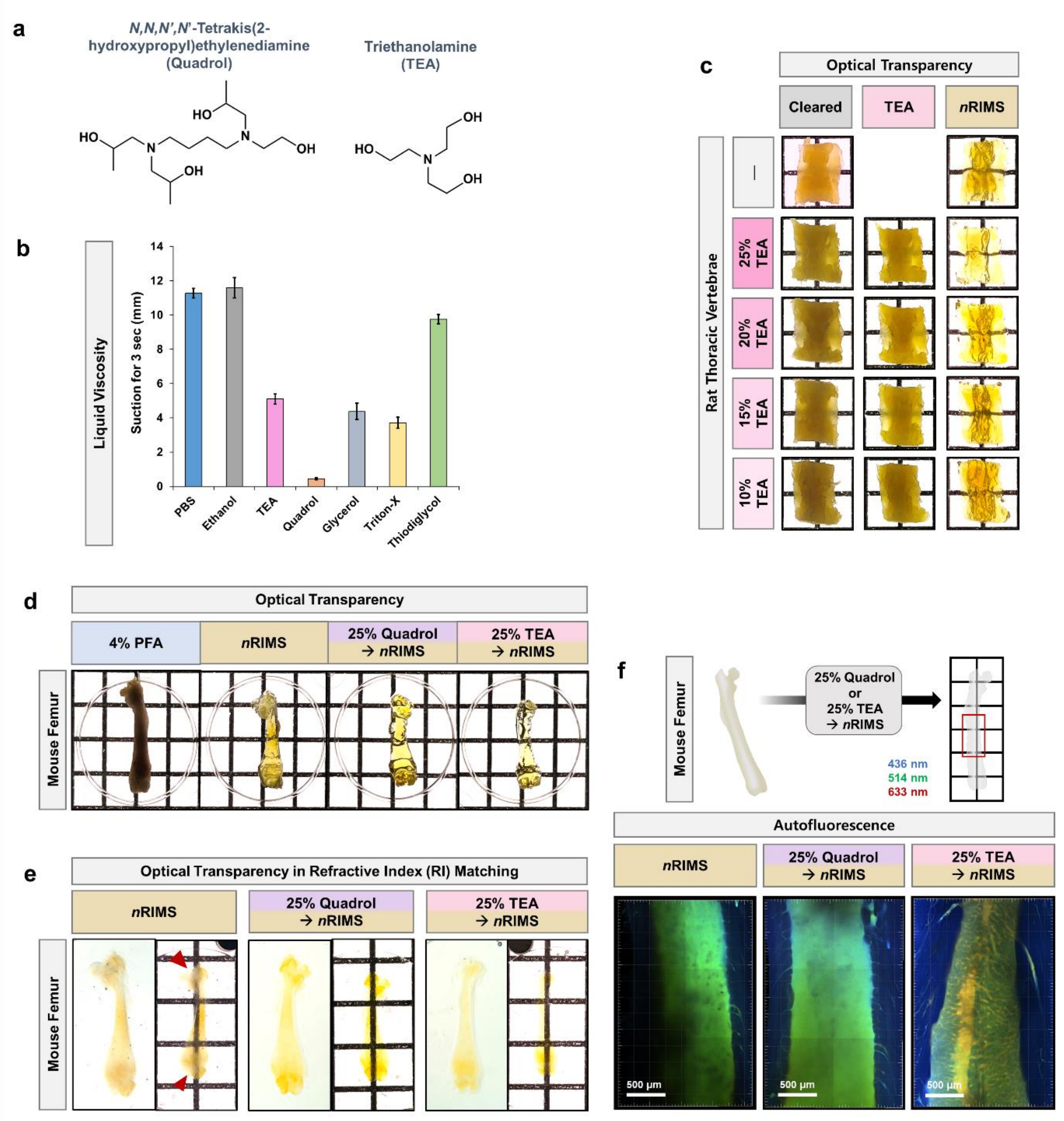

Supplementary Fig. 2. Generation of transparent mouse bones via Bone-mPACT for rapid clearing.

(a) Molecule *N,N,N',N'*-tetrakis(2-hydroxypropyl)ethylenediamine (quadrol) and 25% triethanolamine (TEA) reagents. **(b)** Comparison of the liquid viscosity of PBS, ethanol, TEA, quadrol, glycerol, triton-X and thiodiglycol. **(c)** Comparison of optical transparency images in rat thoracic vertebrae achieved with 10–25% TEA incubation and refractive index matching. **(d and e)** Comparison of optical transparency images

in mouse femurs achieved with 25% quadrol and 25% TEA incubation and refractive index matching with Bone-mPACT+ of Calci-Clear Rapid. The transparency of all cleared samples was evident against a patterned background (length × width = 5 × 5 mm). **(f)** Comparison of autofluorescence images in mouse femur bone achieved with Bone-mPACT+ of Calci-Clear Rapid. Merged images with autofluorescence in green (514 nm), red (633 nm), and blue (436 nm). Scale bar (white: 500 μm).

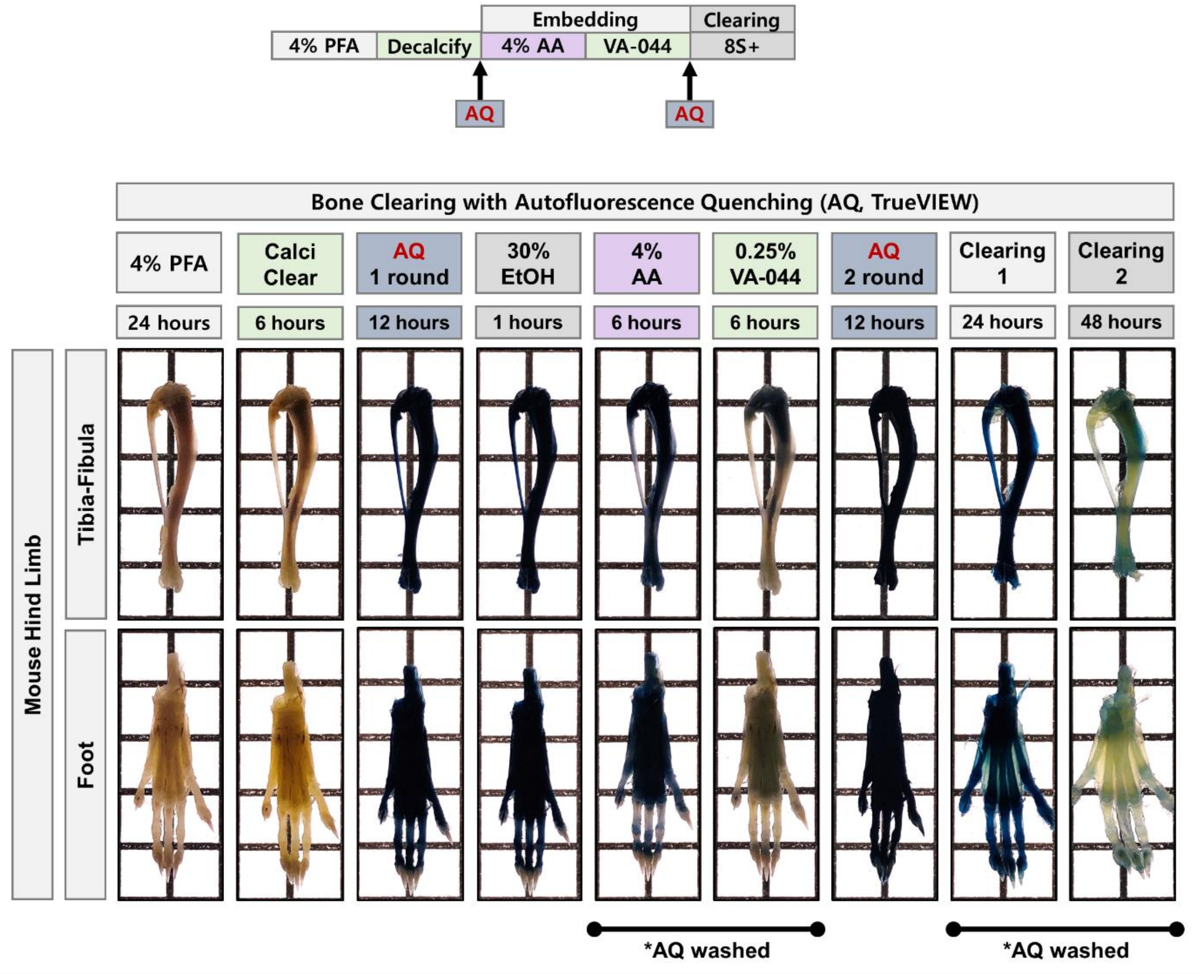

Supplementary Fig. 3. Generation of transparent mouse bones with the bone clearing with autofluorescence quenching.

Comparison of optical transparency in the mouse hind limb bones (tibia-fibula and foot) via bone clearing with Calci-Clear Rapid, using autofluorescence quenching manual (TrueVIEW). Sample were incubated in AQ mixture at each two stages; at after calcification, and at after hydrogel tissue embedded. The transparency of all cleared samples was tested against a patterned background (length × width=5 × 5 mm).

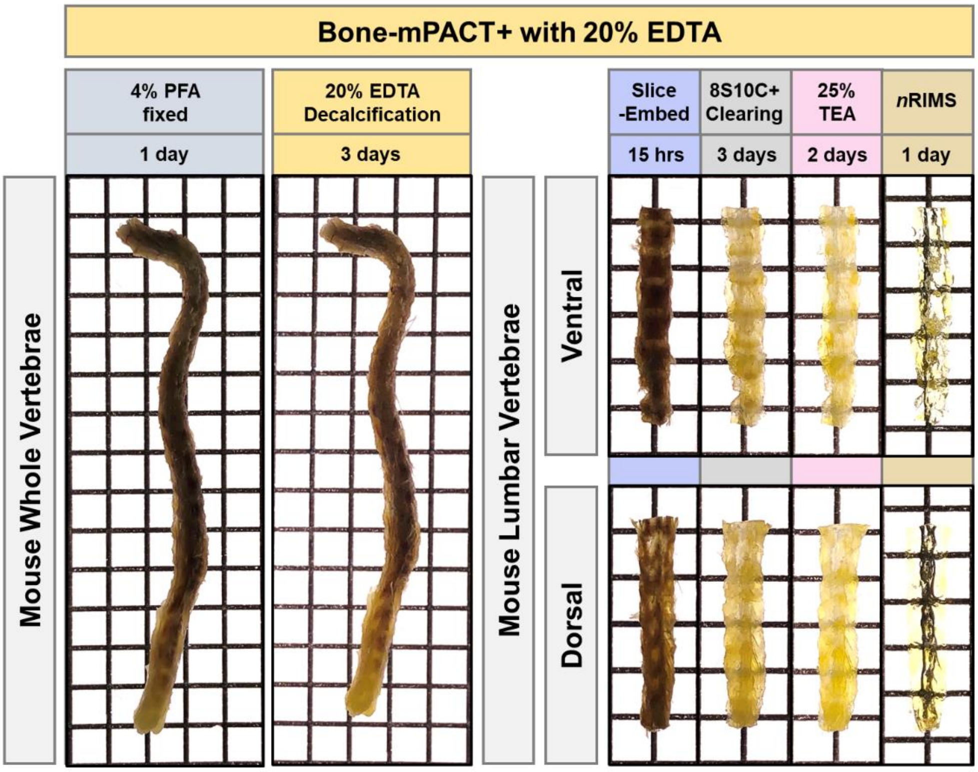

Supplementary Fig. 4. Generation of transparent mouse vertebrae bones with Bone-mPACT+ involving 20% EDTA.

Comparison of optical transparency in the mouse lumbar vertebrae (upper: ventral, lower: dorsal) achieved with Bone-mPACT+ involving 20% EDTA. The transparency of all cleared samples was tested against a patterned background (length × width=5 × 5 mm).

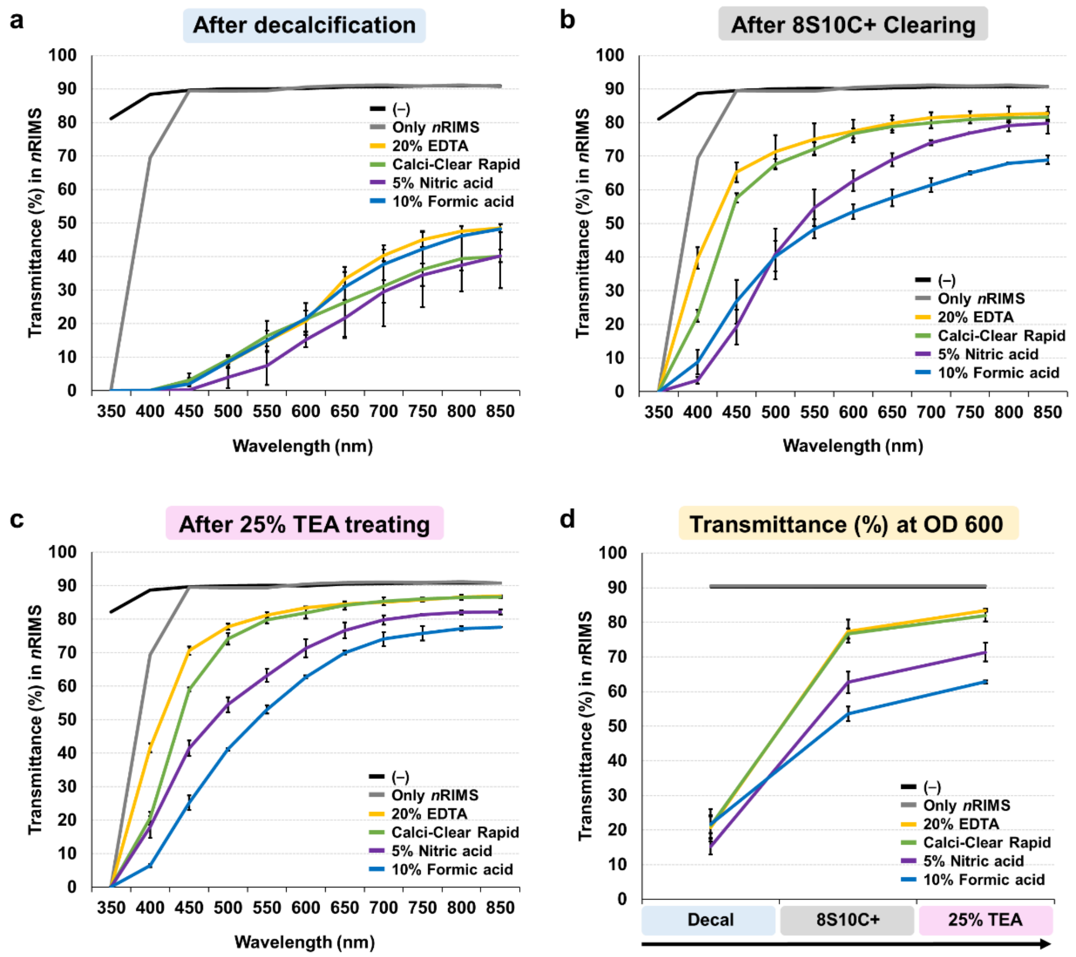

Supplementary Fig. 5. Comparison of bone clearing and transmittance achieved via Bone-mPACT+ with four decalcifications.

Comparison of clearing efficacies of mouse pelvis bone clearing with four decalcification processes and Bone-mPACT+. **(a-c)** Transmittance (%) of mouse pelvis bone after the Bone-mPACT+ bone clearing process with four decalcification solutions (20% EDTA, Calci-Clear Rapid, 5% Nitric acid, and 10% Formic acid). **(d)** Transmittance (%) of cleared mouse pelvis bone at an optical density (OD) of 600 nm. Each color line indicates the assessment values of empty (black) and refractive index matched solutions (*n*RIMS; gray) for each protocol, and four decalcification solutions: 20% EDTA (yellow), Calci-Clear Rapid (green), 5% nitric acid (violet), and 10% formic acid (blue). The results are the averages of five separate tests.

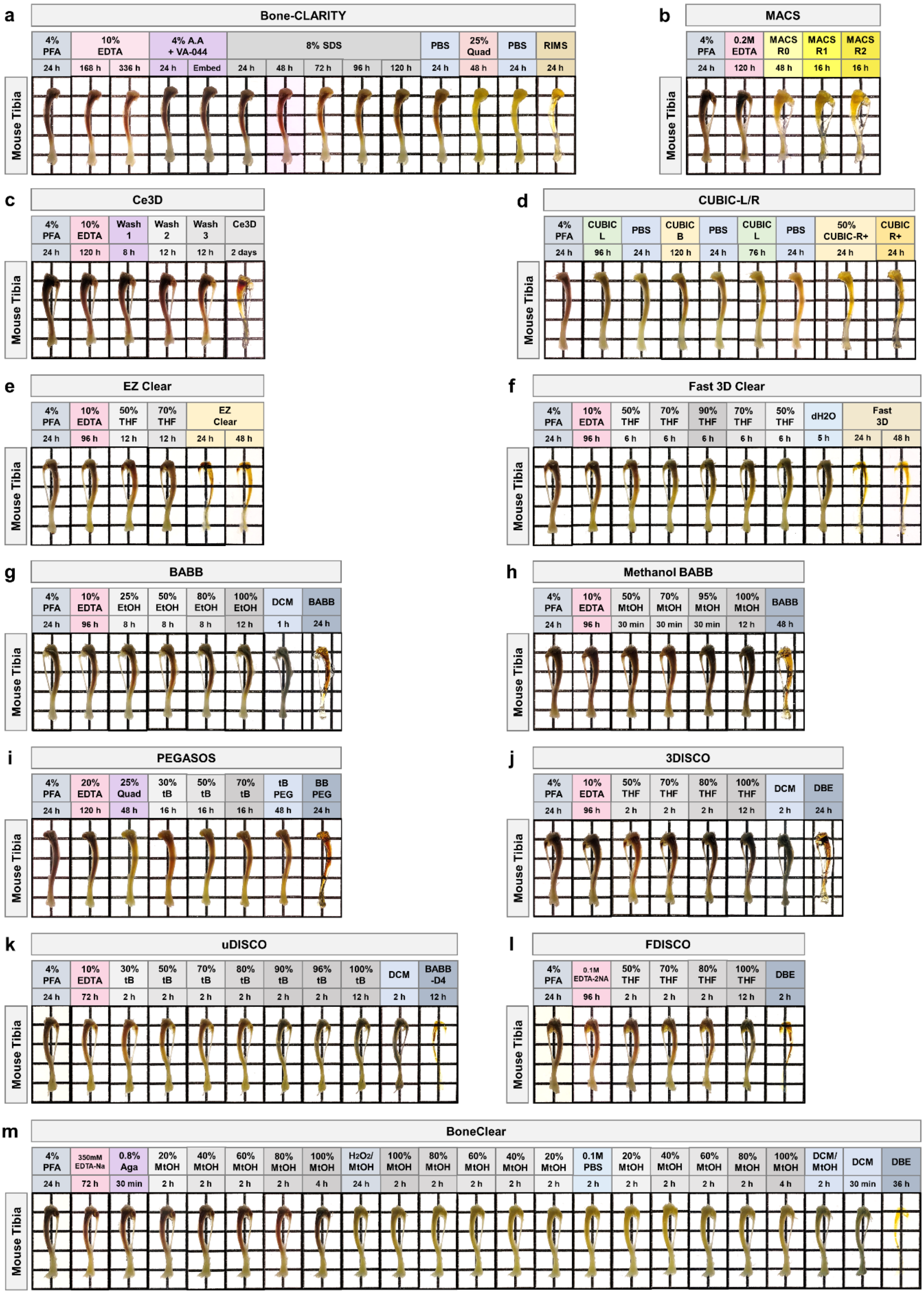

Supplementary Fig. 6. Comparison of tissue clearance process achieved via various clearing methods in mouse tibia.

Comparison of optical clearing process of Bone-CLARITY **(a)**, MACS **(b)**, Ce3D **(c)**, CUBIC-L/R **(d)**, EZ Clear **(e)**, Fast 3D Clear **(f)**, BABB **(g)**, Methanol BABB **(h)**, PEGASOS **(i),** 3DISCO **(j)**, uDISCO **(k)**, FDISCO **(l)**, and BoneClear **(m)** on mouse tibia samples. The transparency of all cleared samples was tested against a patterned background (length × width=5 × 5 mm).

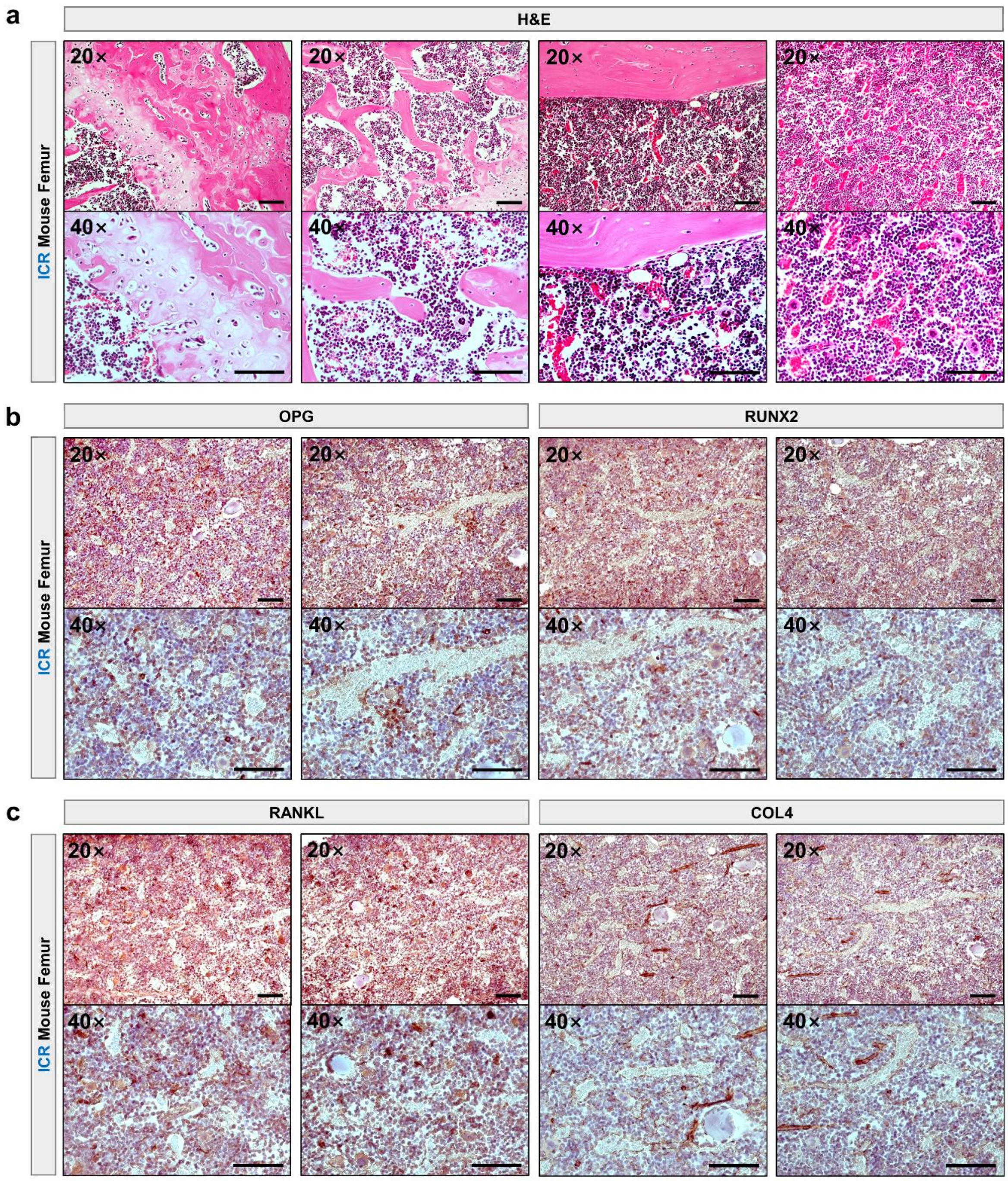

Supplementary Fig. 7. IHC isotype antibody control staining.

Representative images of haematoxylin and eosin (H&E) staining **(a),** and isotype antibody control staining to demonstrate specificity of staining **(b and c)** for OPG, RUNX2, RANKL, COL4. Images show trabecular bone region of mouse femur. Scale bar (black): 1000 μm

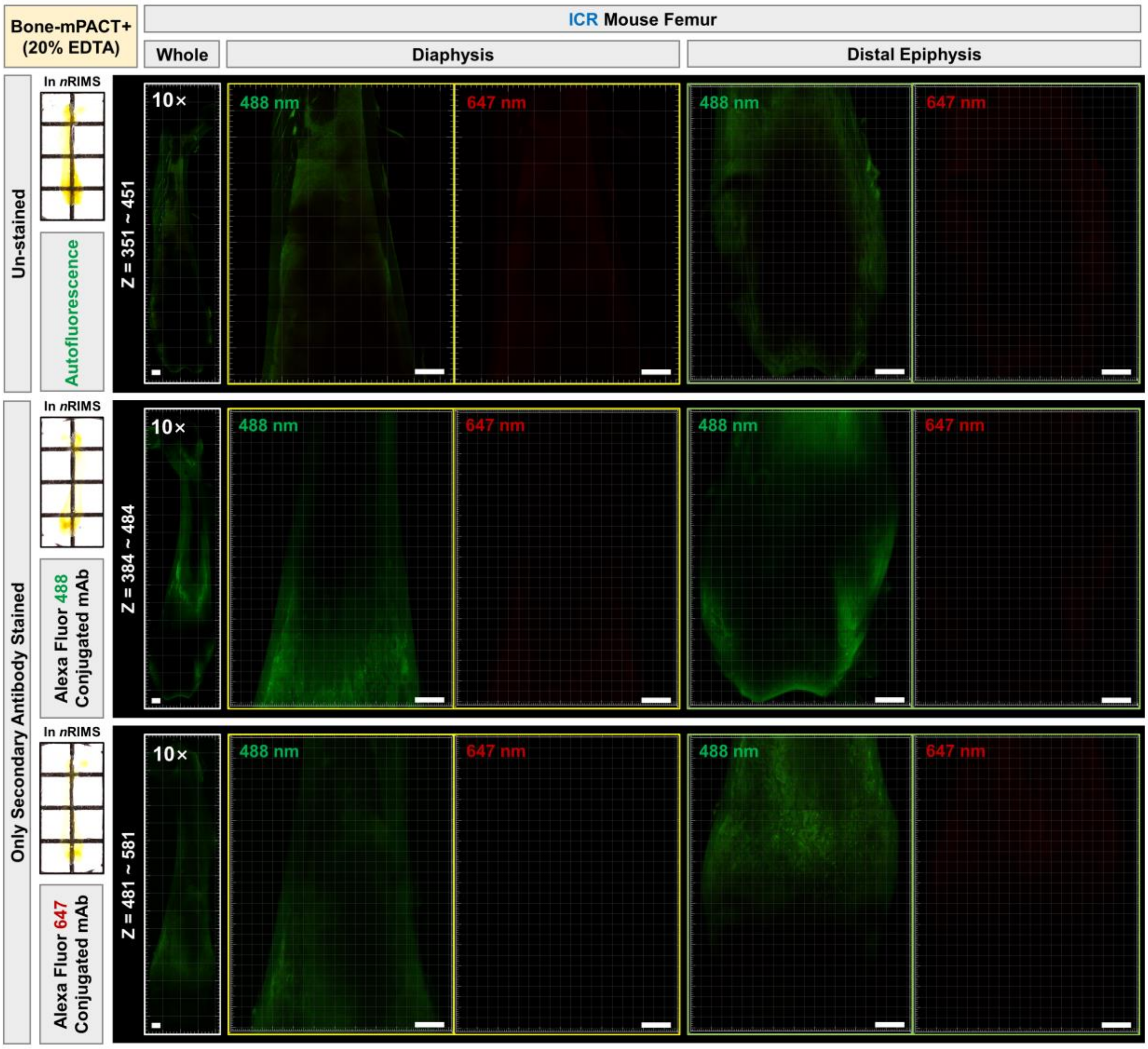

Supplementary Fig. 8. Secondary antibody control staining of mouse femur bone using Bone-mPACT+.

Representative mouse femur bone images of un-stained or single-stained with Alexa Fluor 488 and 647 conjugated secondary mouse antibodies (mAb). All images were acquired with a 10× objective at 488 nm (green) and 647 nm (red) wavelength on a confocal laser microscope. A 3D projection of mouse femur bone (3 × 12 tiled), focusing on the diaphysis (3 × 3 tiled, range: 100 μm) and distal epiphysis (3 × 3 tiled, range: 100 μm) in the femur, including the bone marrow. Scale bar (white: 500 μm).

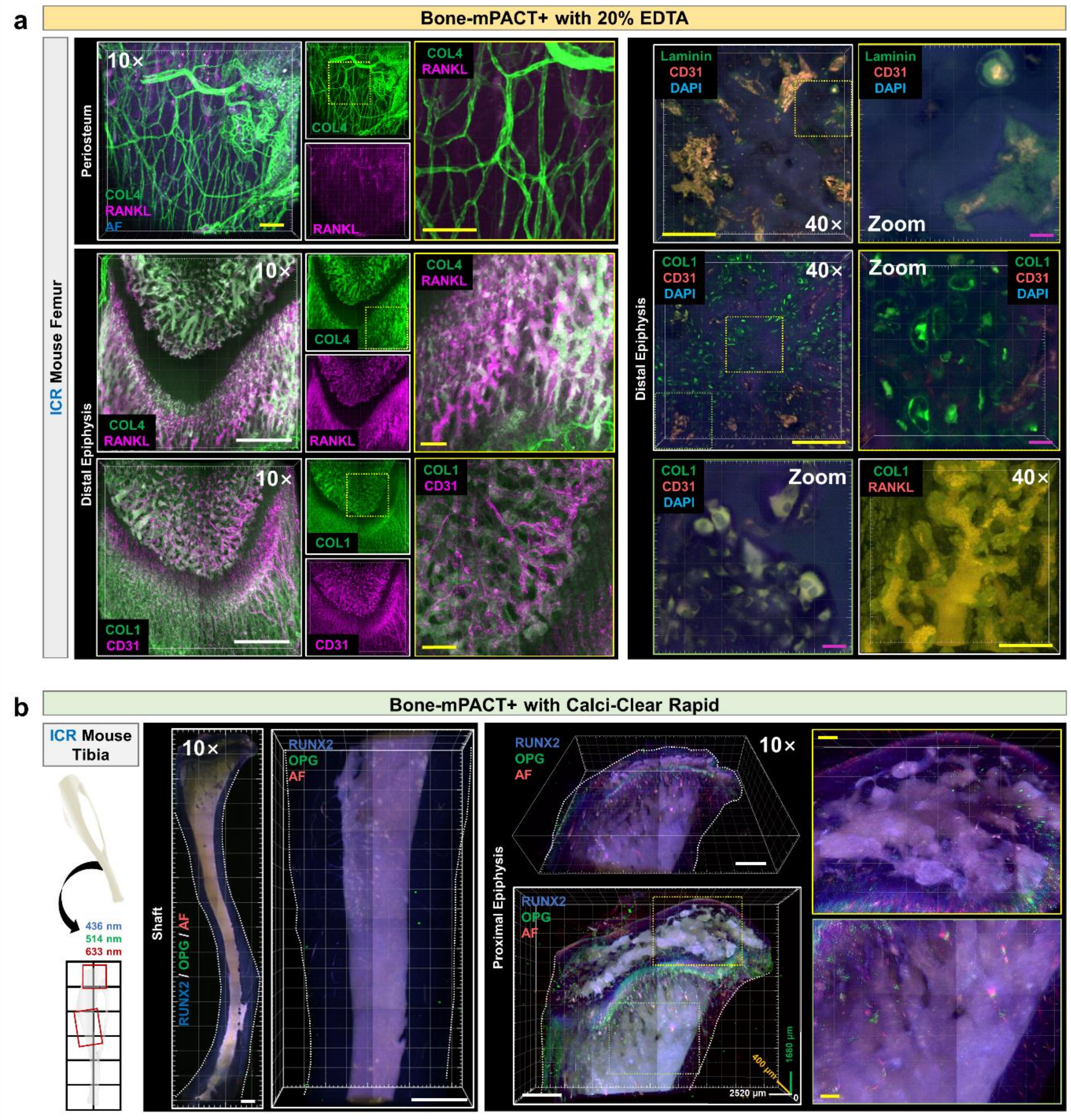

Supplementary Fig. 9. Imaging analysis of mouse bone using Bone-mPACT+. (a) Immunostaining in mouse femur bone processed using Bone-mPACT+ with 20% EDTA. (Left) Images of Collagen type 4 (COL4, green), Collagen type 1 (COL1, green), RANKL (magenta) and CD31 (magenta) immunostaining, and autofluorescence (AF, blue), focusing on the periosteum and distal epiphysis in the femur bone processed using Bone-mPACT+ plus 20% EDTA. (Right) Laminin (green), COL1 (green), CD31 (red), RANKL (red) and DAPI (blue) immunostaining, focusing on the distal epiphysis

in mouse femur bone processed using Bone-mPACT+ plus 20% EDTA. **(b)** OPG (green) and RUNX2 (blue) immunostaining with autofluorescence (AF, red) in mouse tibia bone processed using Bone-mPACT+ plus Calci-Clear Rapid. (Left) Whole endogenous fluorescence images of the tibia (3 × 20 tiled, z=390 μm), (Right) 3D projection of the RUNX2 and OPG expression focusing on the shaft diaphysis (4 × 8 tiled, range: 300 μm), and proximal epiphysis (5 × 6 tiled, range: 800 μm), including the plane regions in the tibia. Autofluorescence is preserved in the bone marrow. All images were acquired with a 10× and 40× objective on a confocal laser microscope. Scale bar (white: 500 μm, yellow: 100 μm, magenta: 10 μm).

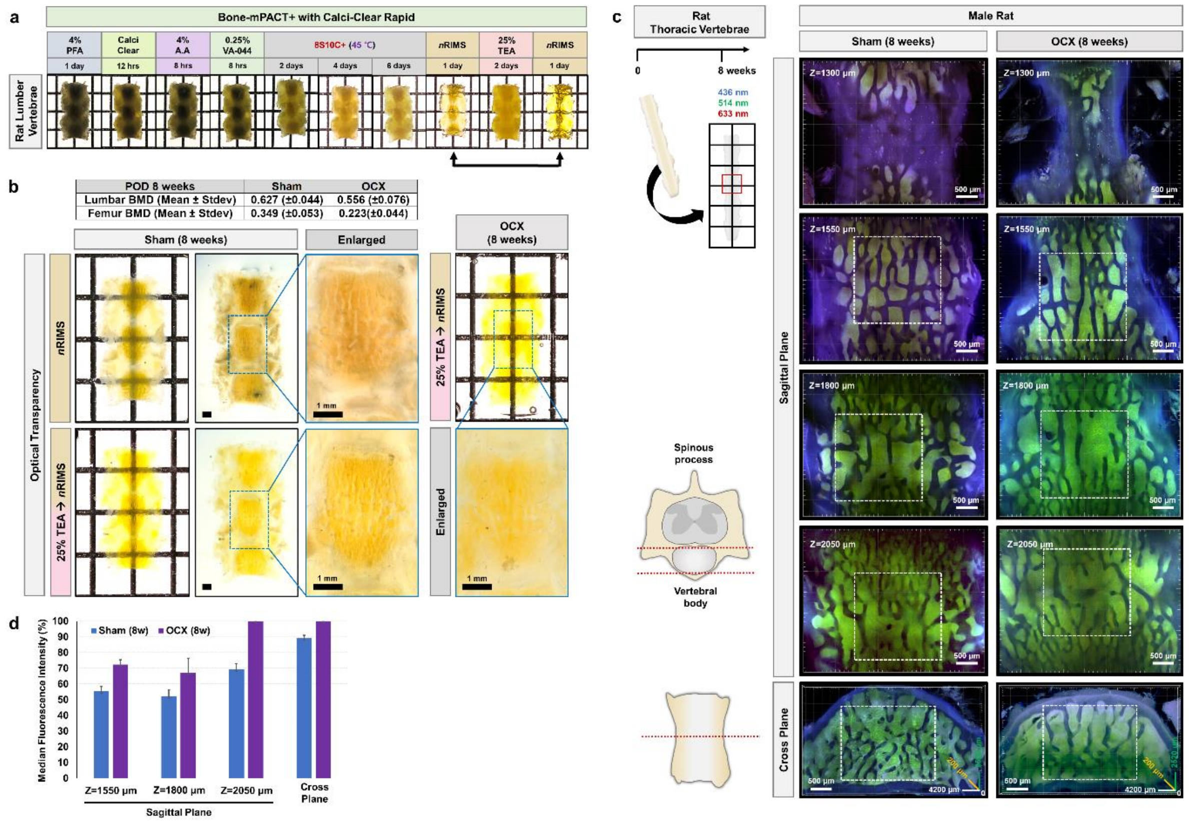

Supplementary Fig. 10. Endogenous fluorescence detection in transparent vertebrae of male osteoporosis model rats.

(a) Comparison of optical transparency achieved in rat lumbar vertebrae using Bone-mPACT+ involving Calci-Clear Rapid. **(b)** Comparison of optical transparency achieved in the rat lumbar vertebral column (L2) of sham-operated rats and orchiectomized (OCX) rats 8 weeks after the operation. Samples were processed with Bone-mPACT+ involving Calci-Clear Rapid. The transparency of all the cleared samples was tested against a patterned background (length × width = 5 × 5 mm). Scale bar (black: 1-mm). **(c)** Comparison of endogenous fluorescence images in the transparent rat lumbar vertebral column (L2) of sham-operated and orchiectomized (OCX) rats 8 weeks after the operation. Merged images showing autofluorescence with three-channel laser light, such as green (wavelength: 514 nm), red (wavelength: 633 nm), and blue (wavelength: 436 nm). Scale bar (white: 500 μm). **(d)** Relative

fluorescence intensity (bone marrow) as a function of images of lumbar vertebral column (L2) **(c)**. Each color line points to assessment values of distinct depth (z-stack: 1500 µm, 1800 µm, and 2050 µm) of the bones (each n=5). The results are the averages of five separate tests.

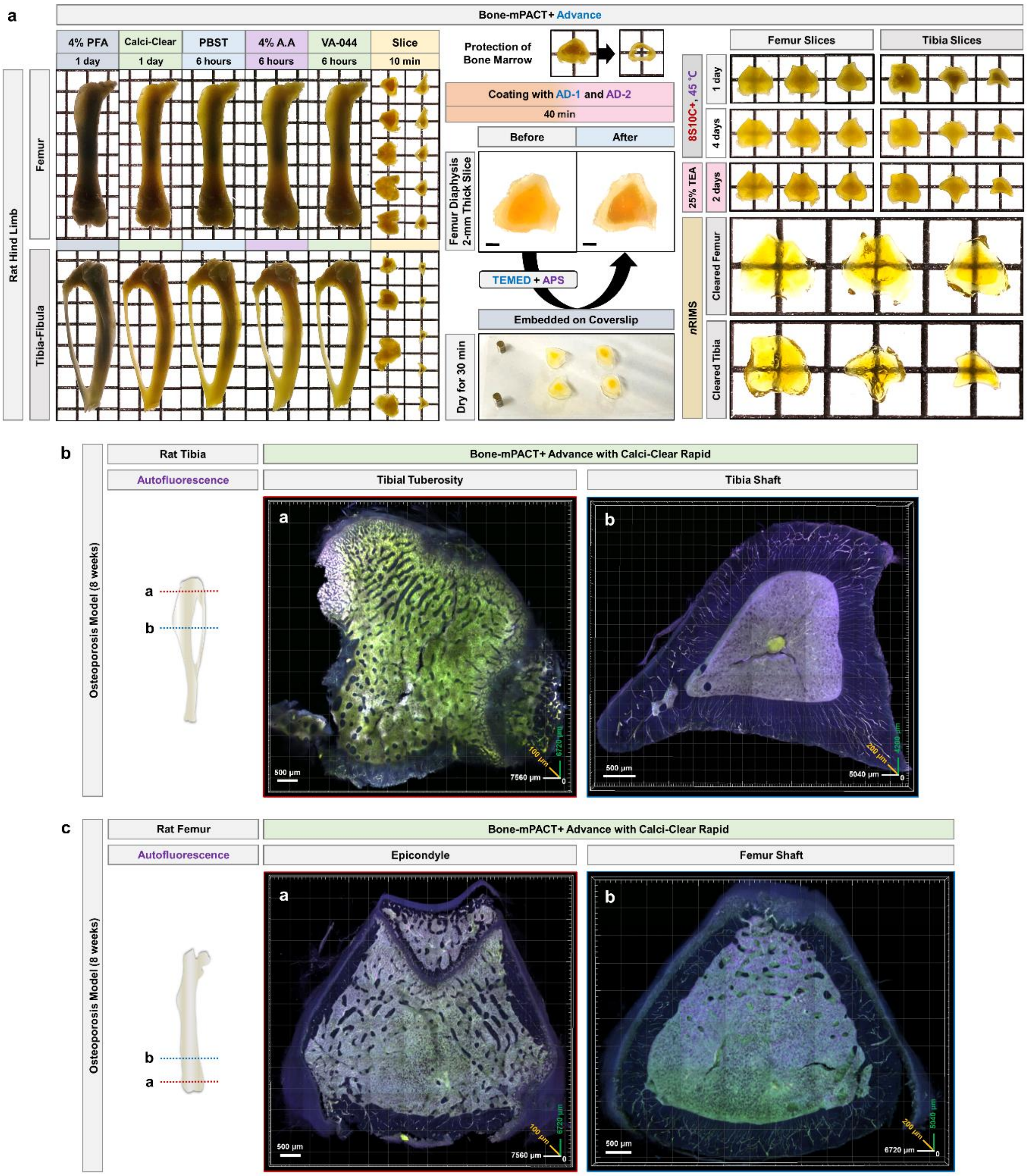

Supplementary Fig. 11. Bone-mPACT+ Advance for large bone.

(a) Comparison of optical transparency achieved in rat femur and tibia bones using Bone-mPACT+ Advance involving Calci-Clear Rapid. The transparency of all cleared samples was evident against a patterned background (length:width=5 mm:5 mm). **(b and c)** High-resolution autofluorescence images of rat tibia and femur bones of orchiectomized rats 8 weeks after the operation using Bone-mPACT+ Advance

involving Calci-Clear Rapid solution. **(b)** Endogenous fluorescence images of the tibial tuberosity (a; 9 × 8 tiled, range: 100 μm) and shaft (b; 6 × 5 tiled, depth: 200 μm) in the transparent rat tibia of orchiectomized rats 8 weeks after the operation. **(c)** Endogenous fluorescence images of the epicondyle (a; 9 × 8 tiled, range: 100 μm) and shaft (b; 8 × 6 tiled, range: 200 μm) in the transparent rat femur of orchiectomized rats 8 weeks after the operation. Merged images with autofluorescence in green (wave length: 514 nm), red (wave length: 633 nm), and blue (wave length: 436 nm). Scale bar (white: 500 μm).
